# Resistance to Powdery Mildew in Qingke Involves the Accumulation of Aromatic Phenolamides Through Jasmonate-Mediated Activation of Defense-Related Genes

**DOI:** 10.3389/fpls.2022.900345

**Published:** 2022-06-30

**Authors:** Congping Xu, Chuansong Zhan, Sishu Huang, Qijun Xu, Tang Tang, Yulin Wang, Jie Luo, Xingquan Zeng

**Affiliations:** ^1^State Key Laboratory of Hulless Barley and Yak Germplasm Resources and Genetic Improvement, Lhasa, China; ^2^Tibet Academy of Agricultural and Animal Husbandry Sciences Lhasa, Tibet, China; ^3^College of Tropical Crops, Hainan University, Haikou, China; ^4^Sanya Nanfan Research Institute of Hainan University, Hainan Yazhou Bay Seed Laboratory, Sanya, China; ^5^Wuhan Metware Biotechnology Co., Ltd., Wuhan, China; ^6^Plant Sciences College, Tibet Agriculture and Animal Husbandry University, Tibet, China

**Keywords:** powdery mildew (*Blumeria graminis* f. sp. hordei), metabolomics, transcriptome, proteome, jasmonic acid, H3K27me3

## Abstract

Powdery mildew (PM) leads to severe yield reduction in qingke (*Hordeum vulgare* L. var. *nudum*). Although studies have focused on identifying PM-related resistance genes, mechanistic insights into the metabolic regulation networks of resistance against PM have rarely been explored in qingke. Here, we integrated transcriptomic, proteomic and metabolomic data using PM-susceptible (G72) and PM-resistant (K69) accessions to systemically explore the mechanisms of PM resistance. The integrated results show that a rapidly transduction of jasmonic acid (JA) and (+)-7-iso-jasmonoyl-L-isoleucine (JA-Ile), and importantly, a inducing accumulation of aromatic PAs conferred qingke-specific resistance for PM stress. Functional analysis revealed that the four BAHD *N*-acyltransferase genes were responsible for the synthesis of aliphatic and aromatic PAs. The expression of the four genes are induced by methyl jasmonate (MeJA) and PM treatment. Co-expression network analysis shows that a histone lysine demethylase, JMJ705 gene, also induced by MeJA and PM treatment, had highly correlation with PAs biosynthesis. Chromatin immunoprecipitation (ChIP)-seq assays revealed that the level of trimethylated histone H3 lysine 27 (H3K27me3) of the four genes in MeJA and PM-treated plants was significantly reduced. Overall, our results suggest that a novel strategy for jasmonic acid signal-mediated demethylation controlling the accumulation of aromatic PAs to enhance plant immune resistance through removal of H3K27me3 and activating defense-related gene expression.

## Introduction

Barley (*Hordeum vulgare* L.) was one of the earliest domesticated and cultivated crops under both natural and artificial selection 10,000 years ago (Badr et al., 2000). Tibetan hulless barley (*Hordeum vulgare* L. var. *nudum*), also called “naked barley” or “qingke” in Chinese, has been used as a principal staple food in the Tibetan area. It is also widely used as livestock feedstuff and raw material for the production of beer, medicine and health care products (Zeng et al., 2015, 2020b). However, the production and quality of Tibetan hulless barley are often challenged by a devastating PM disease caused by the biotrophic pathogen *Blumeria graminis* (DC.) f. sp. *hordei* (*Bgh*) (Piechota et al., 2019). Therefore, a comprehensive understanding of metabolic regulation networks of resistance against PM is essential to explore the underlying mechanisms of PM resistance in qingke.

Extensive research in recent decades has shown that the metabolic network of plants is reprogrammed in response to abiotic and biotic stresses to maintain metabolic homeostasis and the synthesis of various defense-related secondary metabolites, such as plant hormones and flavonoids, to mitigate the adverse effects resulting from stress (Balmer et al., 2013). Recent research has identified that various types of metabolites in qingke, such as phytohormones, phenylpropanoids, lipids, flavonoids and PAs, differentially accumulated and are involved in the plant defense response under PM infection (Yuan et al., 2018). In addition, reports have shown that JA and its derivatives (jasmonates, JAs) have important functions in plant responses and defenses against biotic stresses (Abdollah et al., 2014). Endogenous JA significantly improved plant resistance to fungal pathogen infections, including PM infection (Peng et al., 2012; Abdollah et al., 2014). However, the relationship between JA levels and the defense-related metabolites under biotic stress is still largely unknown.

Technical breakthroughs in next-generation sequencing and metabolomics detection as well as multiomics tools offer many new clues for understanding the molecular mechanisms of metabolic regulation associated with biotic stress responses (Sheikh et al., 2020). A previous study identified a total of 831 significant differentially expressed genes and three pathways using the next-generation RNA sequencing technology, including photosynthesis, plant-pathogen interaction and photosynthesis-antenna proteins that are involved in the response to PM stress in resistant qingke (Zeng et al., 2014). Recently, transcriptomic or metabolomic data were investigated the interaction of PM with many plants, including qingke (Yuan et al., 2018), barley (Li Y. et al., 2019; Brugger et al., 2021), and wheat (Xin et al., 2012; Zhang et al., 2014; Fu et al., 2016). To date, many studies have integrated transcriptomic, metabolic and proteomic data to investigate the global metabolic regulatory networks of gene expression and metabolic regulation in response to PM stress.

Here, to screen potential gene, protein and metabolite regulatory networks associated with qingke resistance to PM, we performed a comparative analysis in resistant (K69) and susceptible (G72) qingke accessions using the integration of transcriptomic, metabolomic and proteomic data. Through a combination of multiomics analyses, we constructed a coexpression network to control the accumulation and synthesis of important metabolites in response to PM infection. In addition, we provided exogenous *N*-feruloyl serotonin and *N*-feruloyl tyramine on leaves of the G72 line to confirm the importance of plant antifungal metabolites during the resistance response against PM. This underlying mechanism researched in this study may be used to increase PM resistance during crop genetic breeding improvement programs.

## Materials and Methods

### Plant Material and Powdery Mildew Infection

Two cultivated qingke accessions, one resistant (K69) and one susceptible (G72), were identified from the core germplasm resources of qingke based on previously published methods (Yuan et al., 2018). Two qingke accessions were grown at the Tibet Academy of Agricultural and Animal Husbandry Sciences, Lhasa, Tibet Province, China. Twenty-day-old seedlings in plastic pots of two accessions were infected with the *Bgh* race (Yuan et al., 2018) and the disease phenotype and symptoms were monitored at 6, 36, 72 and 168 hpi. Plants were grown in a greenhouse at 25°C with a 14 h light/10 h dark photoperiod. Leaf tissues were collected at 6, 36, 72, and 168 hpi, frozen immediately in liquid N_2_ and stored at −80°C. Subsequent experiments were carried out with three biological replications for each treatment point.

### Disease Phenotype Investigation and Enzyme Assay

Minor modifications were performed in a previous study (Luo et al., 2021) to investigate the disease incidence and index in two qingke accessions under 6, 36, 72, and 168 hpi. Six physiological indices were measured with three biological and technical replicates in two qingke lines under 6, 36, 72, and 168 hpi. Leaf photosynthesis was measured using a Li6400 porData photosynthesis system (Li-COR Biosciences, Lincoln, NE, United States) with a 2 × 3 cm leaf cuvette. The plant height of the two accessions was measured using a ruler of 10 seedlings for each replicate. The extraction of antioxidant enzymes, including catalase (CAT), superoxide dismutase (SOD), peroxidase (POD) and the malondialdehyde (MDA) content, was performed according the method described in Zhang et al. (2015).

### RNA Extraction and Sequencing Analysis

Total RNA was isolated from the ground leaf samples at the specified time points (0, 6, 36, 72, and 168 hpi) using the RNA Nano Assay Kit (Agilent Technologies, Santa Clara, CA, United States) according to the manufacturer’s instructions. Libraries were constructed and performed on the DNBSEQ platform. Clean reads were filtered from raw reads by removing of low-quality reads and adaptors and ambiguous reads using SOAPnuke (v1.4.0) using the following parameters:-l 15 -q 0.2 -n 0.1. High-quality clean reads were assembled and blasted against the National Center for Biotechnology Information (NCBI) non-redundant nucleotide database (nt) and mapped to the improved qingke genome (Zeng et al., 2020a) using HISAT2 (version 2.1.0^1^) with the following parameters: –dta –phred64 unstranded –new-summary -x index -1 read_r1-2 read_r2 (PE) (Kim et al., 2015). Alignment output files were assembled and transcripts were generated using Cufflinks version 2.2.0. The relative expression of transcripts was estimated based on the number of fragments per kilobase of exon per million mapped reads (FPKM) using HTSeq 0.12.4 (Trapnell et al., 2010; Anders et al., 2015). Identification of differentially expressed genes (DEGs) in both lines at different treatment points was performed and generated using the DESeq Package v1.12.0 (*p* < 0.05; log_2_fold change (FC) > 1 or <−1). Gene ontology (GO) terms and Kyoto Encyclopaedia (KEGG) pathway analysis of DEGs were mapped by the GO Term Finder^2^ and KEGG Orthology databases (Kanehisa et al., 2007). The clean data were submitted to the NCBI BioProject database (accession number PRJNA728483).

### Detection and Analysis of Metabolites

Leaves of two qingke accessions at five time points (0, 6, 36, 72, and 168 hpi) after PM infection were selected for metabolite analysis using a LC-ESI-MS/MS system (HPLC, Shim-pack UPLC SHIMADZU CBM30A system; MS, 6500 Q TRAP). In addition, leaves of two PM-resistant accessions, including one qingke line K69 and one cultivated hulled barley line WDM00496 (termed barley hereafter), at two time points (0 and 24 hpi) after PM infection were also selected for metabolite analysis. These freeze-dried samples were extracted and qualified as previously described (Zeng et al., 2020b). Metabolites with | log_2_FC| ≥ 1 and variable importance (VIP) were identified as differentially accumulated metabolites (DAMs) and were used for KEGG pathway enrichment analysis by the Metware (Wuhan, China) online analytic website^3^.

### Proteome Profiling and Data Analysis

Proteins were extracted as previously described (Wang et al., 2016). Two grams of leaves of two qingke accessions (K69 and G72) at three time points (0, 36, and 168 hpi) was ground to powder with a heavy pestle in liquid nitrogen and transferred to the extraction solution [100 mM Tris-HCl, 1 mM ethylenediaminetetraacetic acid (EDTA), 0.1% Triton-X-100, 10 mM dithiothreitol (DTT)]. The mixture was placed at −2°C for 12 h. The supernatant was obtained and subjected to protein analysis. The isobaric tag for relative and absolute quantitation (iTRAQ) technique and Q-Exactive HF (Thermo Fisher Scientific, Scan Jose, CA) were used for quantification of protein abundance. The raw MS/MS data were searched from the improved qingke database (Zeng et al., 2020b), and standard proteins were identified with two software tools-Proteome Discoverer (version 2.1.1.21) and Mascot (version 2.3)—according to the method described previously with minor modifications (Zhang et al., 2021). Differentially expressed proteins (DEPs) were identified under the following criteria [FC ≥ 1.5 or FC ≤ 0.67, *p* ≤ 0.05]. KEGG pathway analysis of DEPs was implemented using KOBAS 2.0 software (Xie et al., 2011).

### Exogenous Aromatic Phenolamides Treatment

Three-week-old seedlings of the G72 line in plastic pots were infected with the *Bgh* race and then sprayed with a solution containing 0, 15, 30 and 60 mM *N*-feruloyl tyramine or *N*-feruloyl serotonin. Furthermore, a combination treatment of 30 mM *N*-feruloyl tyramine and 30 mM *N*-feruloyl serotonin was also added to this experiment. To ensure the best efficiency of the spray treatments, we continued to perform sprays every day until 10 days after later collecting the sample. For each treatment, three biological replications were performed. These samples were rapidly frozen with liquid nitrogen and then stored at −80°C for further analysis.

### RT–PCR Analysis and Validation

Total RNA was extracted and purified from samples using an RNA extraction kit following the manufacturer’s instructions. Real-time RT–PCR was performed on a SYBR Green system (TaKaRa, Dalian, China). The cycling conditions were 5 min of predenaturation at 95°C, then 94°C for 30 s, 56°C for 30 s, and 72°C for 90 s (35 cycles). The pair of housekeeping genes (*HvACT3*:*HOVUSG4775200* + *HvUBQ1*:*HOVUSG1981000*) was used as an internal control to calculate relative gene expression in this study. The CT for the internal control was the geometric mean of the two housekeeping gene Ct values. The gene expression for each sample was measured by RT–PCR using the relative quantification method (Sébastien et al., 2014). Primers were designed using Beacon Designer™ 7.9 software (Premier Biosoft, San Francisco, United States) and are listed in Supplementary Table 1. All cases were performed on three independent biological and technical repeats.

### Methyl Jasmonate Treatment

Two-week-old seedlings of the K69 line grown in a greenhouse were sprayed with 200 μM MeJA. After 24 h of treatment, leaves were collected and stored at −80°C for later processing.

### Constructing a Metabolic Pathway Network Using Integrating Multiomics

Pearson’s correlation coefficient (PCC) algorithm was used to construct the regulatory networks of three Omics by R software (Bishara and Hittner, 2012). The PCC method is widely accepted and used as a measure of correlation in multiomics analysis. A rigorous multiple test correction (PCC > 0.6) was used to filter the correlation coefficient from two sets of expression values for two entities (inter- or intra-omics data). Cytoscape software (version 3.6.1) was used for the network visualizing all associations among genes, proteins and metabolites (Kohl et al., 2011). Nodes denoted by triangles, diamonds and ovals represent transcripts, proteins and metabolites, respectively. Transcripts, enzymes and metabolites can be presented in the context of the correlation network.

### Phylogenetic Analysis

MEGA 7.06 software was used for the construction of a neighbor-joining tree. Bootstrap scores (1,000 replicates) in all branches are shown as percentages. The amino acid sequences in this study were aligned using Genedoc software and are listed in Supplementary Table 2.

### Recombinant Protein Analysis and Enzyme Activity Assays

The full-length complementary DNAs of five candidate genes from qingke were cloned into the pGEX-6p-1 prokaryotic expression vector (Novagen, Darmstadt, Germany) with glutathione *S*-transferase and transformed into the BL21 (DE3) strain (Novagen). All recombinant proteins were purified and verified as previously method described (Peng et al., 2017).

The *in vitro* enzyme reactions for five candidate genes were measured at 30°C in a 100 μl solution containing 200 μM amide acceptor, 100 μM acyl substrates, 500 ng of the purified recombinant protein and 10 mM MgCl_2_ in Tris-HCl butter (pH 7.4) buffer. After incubating for 20 min, 300 μl ice-cold methanol was added to stop the enzyme reaction. The reaction mixture was filtered using a 0.2 μm filter [(Millipore (Shanghai) Trading Co., Ltd., Shanghai, China)] and then analyzed using LC–MS.

### Transient Overexpression of Candidate Genes in *Nicotiana benthamiana*

Transient expression constructs of candidate genes were generated by first directionally inserting the full-length cDNAs into the pDONR207 entry vector and then into the destination vector pEAQ-HT using Gateway recombination technology (Invitrogen) and further sequenced. The right constructs were introduced into *Agrobacterium tumefaciens* (EHA105). Positive clones were selected and grown to an optical density OD600 of 1.8 in 20 ml of Luria–Bertani (LB) medium (5 g/l yeast extract, 10 g/l tryptone, 10 g/l NaCl, 50 μg/ml kanamycin), washed with washing buffer (10 mM 2-(*N*-morpholino) ethanesulfonic acid (MES), pH = 5.6), and resuspended in MMA buffer (10 mM MES (pH = 5.6), 100 mM acetosyringone, 10 mM MgCl2) to an OD600 of 1.0. The culture was incubated for 2 h at 30°C, and 1 ml of culture was used to infiltrate the underside of 1-month-old *Nicotiana benthamiana* leaves. Leaves were collected for infiltration for 6 days and rapidly frozen with liquid nitrogen and stored at −80°C for later analysis. The freeze-dried samples were extracted overnight at 4°C with 1.0 ml 70% aqueous methanol. After centrifugation at 12,000 × *g* for 15 min, the extracts were filtered using 0.22-mm filters (ANPEL^4^, Shanghai, China) before using high-throught-put LC–MS/MS analysis.

### Chromatin Immunoprecipitation and Chromatin Immunoprecipitation-Seq Data Analysis

K69 leaves treated with MeJA, PM and ethanol (mock) were used for ChIP, which was performed mainly as previously described (Zha et al., 2020). The antibodies used for immunoprecipitation reactions with the histone marks were H3K27me3 antibodies (CST9733, CST). Illumina sequencing libraries were constructed and sequenced on the Illumina HiSeq 4000 platform with the PE 150 method by Wuhan IGENEBOOK Biotechnology Co., Ltd.^5^ with three biological replicates for each sample. The ChIP-seq data of nine samples have been deposited in NCBI under the BioProject accession number PRJNA789334. For the ChIP-seq data analysis, low-quality reads were filtered out, and clean reads were mapped to the improved qingke reference genomes (Zeng et al., 2020a). Peaks of four genes were found with MACS2 software (version: 2.1.1.20160309) using the default parameters.

### Chromatin Immunoprecipitation Quantitative Real-Time PCR Validation

Chromatin immunoprecipitation quantitative real-time PCR (Chip-qPCR) assays from MeJA, PM and ethanol (mock) treatment of samples were performed with H3K27me3 antibodies (Millipore, Cat#, 07-449) according to previously described methods with some modifications (Li et al., 2013; Zhan et al., 2020). All ChIP-qPCR assays were performed with three biological replicates.

## Results

### Disease Development of the Resistant (K69) and Susceptible (G72) Qingke Accessions

**FIGURE 1:**
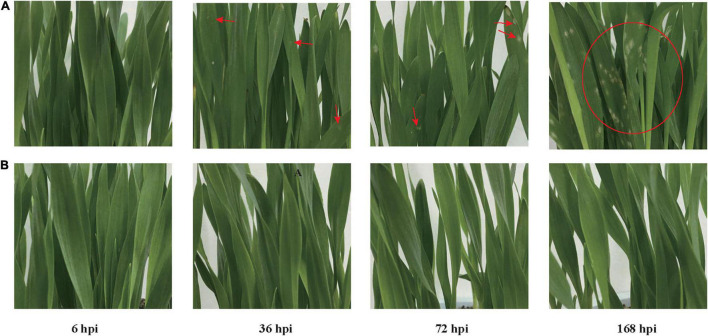
Disease development progression of G72 (one susceptible line to powdery mildew) and K69 (one resistant line to powdery mildew) at 6, 36, 72, and 168 h post-inoculation (hpi). **(A)** G72 line. **(B)** K69 line.

Two cultivated qingke accessions, one resistant K69 (Gannongda69) and one susceptible G72 (Zang0072), were identified from the core germplasm resources of qingke based on previously published methods (Yuan et al., 2018). *Blumeria graminis* (DC.) f. sp. *hordei* (*Bgh*) strain 16248-4 was used to infect plants of G72 and K69 (Yuan et al., 2018). For the G72 line, small lesions appeared on the leaves at 36 hpi. These lesions then expanded quickly and heavily distributed on the leaves at 72 hpi. Constant PM infection led to disease spots eventually covering the whole seedlings at 168 hpi (Figure 1A). In contrast, the rate of disease progression was largely restricted and lesions were appeared in the K69 line until 168 hpi (Figure 1B). The incidence and disease index of PM in the two qingke lines further confirmed that the resistance to PM in the K69 was significantly higher than that in the G72 (Supplementary Figure 1). To better evaluate the influence of PM inoculation on both qingke lines, six physiological indices were measured at 0, 6, 36, 72, and 168 hpi. Plant height and photosynthesis in the K69 line were less affected by PM stress than those in the G72 line. In addition, the activity of antioxidant enzymes, such as POD, SOD and CAT, was higher in the K69 line than in the G72 line during PM infection. However, the K69 line displayed significantly lower MDA content than the G72 line under PM stress (Supplementary Figure 2). Collectively, these results indicated that the K69 line was more resistant to PM infection than the G72 line.

### Differentially Expressed Genes Between K69 and G72 During Powdery Mildew Infection

To investigate the transcriptional regulation response to PM stress in the two qingke lines, we performed a transcriptome analysis of 30 samples. The statistics of the sequencing library of each sample are registered in Supplementary Table 3. Approximately 40 (41.49–45.41) million clean reads were generated, among which nearly 90% (average 86.97%) clean reads could be mapped to the qingke reference genomes (Zeng et al., 2020a) at the five time points during PM treatment. Notably, at 168 hpi, the clean reads mapped to the fungal sequence were significantly higher in the G72 line (6.16%) than the K69 line (1.22%), which suggested extensive colonization of the qingke leaf by PM, particularly in the later stages of the infection process. The differentially expressed genes (DEGs) were screened under PM infection and non-stress control at the five time points in both lines. Subsequently, 6,832, 9,142, 8,308, 7,060 and 10,542 DEGs were identified between the resistant K69 and the susceptible G72 lines at 0, 6, 36, 72, and 168 hpi, respectively (Supplementary Table 4). In addition, only 344 (1.7%) and 465 (2.2%) genes were shared and differently up/down expressed in the K69 line compared to G72 line under normal and PM treatment. It indicates that most of DEGs were caused by the different responses to PM infection in the two qingke lines, rather than the differences in the genetic background of the two lines (Supplementary Figure 3A). To further investigate the pathway involved in resistance to PM, we performed KEGG enrichment analysis of DEGs at 6, 36, 72, and 168 hpi. The results indicated that DEGs at 6 hpi were enriched in plant hormone signal transduction, and DEGs at the other three time points were significantly enriched in flavonoid and phenylpropanoid biosynthesis pathways (Supplementary Figure 4).

### Metabolite Analysis of Resistant and Susceptible Qingke in Response to Powdery Mildew

To explore the metabolic changes of qingke that occur following PM infection, we performed a widely targeted liquid chromatography-tandem mass spectrometry (LC–MS/MS) -based metabolomic analysis with PM-treated qingke leaf samples collected from the K69 and G72 lines at the selected time points (0, 6, 36, 72, and 168 hpi) (Chen et al., 2013). A total of 568 metabolites were identified and quantified, of which 406 metabolites were annotated, including 24 amino acid derivatives, 141 flavonoids, 28 PAs, 26 phenylpropanoids, 24 lipids, 19 organic acids, 30 nucleotides and their derivatives and 114 other metabolites (Supplementary Table 5). A principal component analysis (PCA) based on the level of metabolites showed that 30 sample tissues were clustered into two groups, represented by K69 and G72 (Supplementary Figure 5A). In addition, metabolite accumulation patterns of the susceptible G72 line displayed clearer spatial separation than those of the resistant K69 line under PM stress (Supplementary Figure 5A). Furthermore, 69 (up: 27; down: 42), 152 (up: 102; down: 50), 188 (up: 119; down: 69), 168 (up: 95; down: 73) and 201 (up: 98; down: 103) differentially accumulated metabolites (DAMs) of both lines were identified at 0, 6, 36, 72, and 168 hpi, respectively (Supplementary Table 6). No DAMs were shared in the two varieties under normal and PM treatment, suggesting that all DAMs of both lines were caused by the different responses of the two qingke lines under PM infection, rather than the differences in the genetic background of the two cultivar lines (Supplementary Figure 3B). Furthermore, a KEGG analysis of DAMs at four time points revealed that pathways of plant hormone signal transduction, phenylpropanoid, tryptophan and amino acid biosynthesis, and flavonoid metabolism were significantly enriched at least two time points, which represented core metabolic responses to PM infection (Supplementary Figure 6).

### Proteomic Analysis of Resistant and Susceptible Qingke in Response to Powdery Mildew

The K69 and G72 lines had distinct differences at 36 hpi and 168 hpi by analyses of phenotype, transcriptomic and metabolomic data (Figure 1, Supplementary Figure 2, and Supplementary Tables 4, 6). Therefore, sample tissues of the two qingke lines at both time points and the control (0 hpi) were selected for proteomic analyses. A total of 6,373 proteins were identified and analyzed using iTRAQ (Supplementary Table 7). The PCA score plot based on protein levels clearly distinguished the two qingke lines (Supplementary Figure 5B), which was consistent with the distribution pattern of metabolites (Supplementary Figure 5A). Differentially expressed proteins (DEPs) at 0, 36, and 168 hpi were identified between K69 and G72 (Supplementary Table 8). In addition, 4 DEPs (1.0%) differed between the two varieties in the non-stress control and PM infection, suggesting that these DEPs were due largely to different responses under PM stress rather than differences in the genetic background of the two cultivar lines (Supplementary Figure 3C). The significant DEPs were mainly enriched in plant hormone signal transduction and phenylpropanoid biosynthesis at 36 and 168 hpi through KEGG pathway analyses (Supplementary Figure 7). Taken together, the transcriptomic, metabolomic and proteomic analyses of the two qingke lines indicated that early transduction of plant hormone signals and phenylpropanoid biosynthesis are crucial biological pathways involved in resistance to PM in qingke. Therefore, the differential regulation of genes related to plant hormone signal transduction and the phenylpropanoid pathway is interesting and will be the subject of further exploration.

### Jasmonic Acid Signaling Was Induced During the Early Stages of Powdery Mildew Infection

**FIGURE 2:**
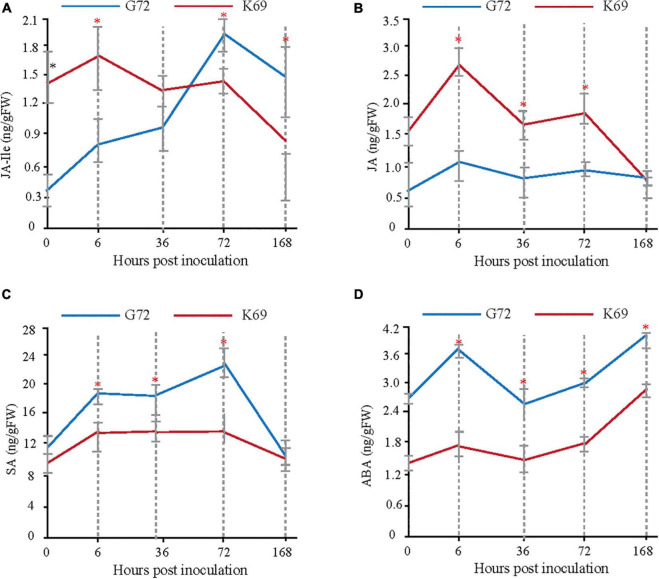
Measure and comparison of phytohormones levels between the K69 and G72 lines during powdery mildew infection. * Indicates significant difference (*P* < 0.05, according to t-test).

Transcriptomic, metabolomic and proteomic analyses indicated that, plant hormone signal transduction is mainly involved in the early response (6 and 36 hpi) to PM infection (Supplementary Figures 4, 6, 7). To determine which plant hormones play important roles in the early plant–pathogen interaction stages of PM treatment, the dynamic changes in abscisic acid (ABA), salicylic acid (SA), JA and JA-Ile were measured in the two qingke lines during PM infection using targeted GC–MS analysis. Different patterns of phytohormone accumulation were identified between the two line treated with PM (Figure 2). JA-Ile and JA levels were rapidly induced at 6 hpi in the K69 line compared to the G72 line. Then, a late significant decrease in JA-Ile and JA was observed in the K69 line, and the values were similar to or lower than that of G72 (Figures 2A,B). However, the level of JA-Ile, a bioactive form of JA, was gradually increased throughout the course of infection (6–168 hpi). Meanwhile, the relative expression level of JA-related genes was obviously up-regulated in the K69 compare to G72 accession under PM infection (Supplementary Table 9). Thus, a longer time to accumulate JA-Ile may reflect a delayed response to fungal colonization in the G72 accession. Further, SA and ABA are working as antagonistics for different plant–pathogen interactions (Wolfgang et al., 2010), and also as critical signaling molecules to promote leaf senescence and cell death (Zhu et al., 2020). In this study, the level of SA and ABA were markedly induced in the G72 line in response to powdery mildew (PM) infection (Figures 2C,D). The results described above showed that resistance to PM stress in qingke was closely associated with a rapid induction of JA signaling. Thus, the timing of this induction seems to be crucial for the activation of defense against this infection.

### The Induced Production of Aromatic Phenolamides Was Responsible for Resistance to Powdery Mildew in Qingke

**FIGURE 3:**
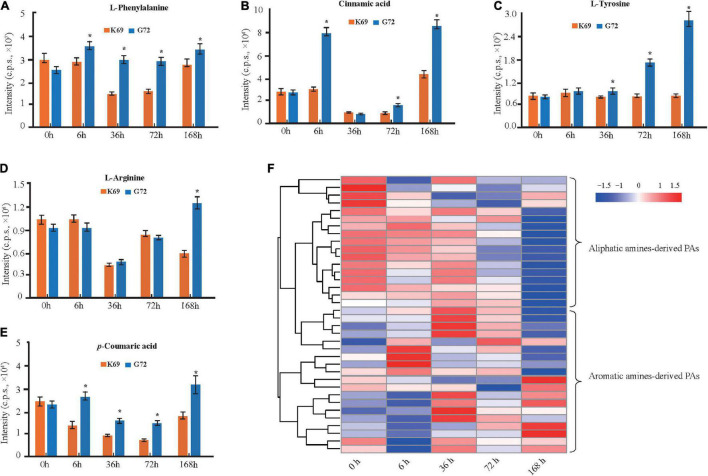
DAMs related to PAs and its precursors were compared in K69 and G72 line during powdery mildew treatment. The levels of PAs precursors **(A)** L-phenylalanine, **(B)** cinnamic acid, **(C)** L-tyrosine, **(D)** L-arginine, and **(E)** *p*-coumaric acid in both of lines. The bars shown as SD with triplicate repeats. * Indicates significant difference (*P* < 0.05, according to *t*-test). **(F)** Heat map visualization of PAs by comparison between K69 and G72 lines under PM infection and the non-stress control at 0, 6, 36, 72, and 168 h time points.

Phenolamides, referred as hydroxycinnamic acid amides, constitute a major group of secondary metabolites resulting from the conjugation of hydroxycinnamic acid derivatives (benzoyl-CoA, cinnamoyl-CoA, *p*-coumaroyl-CoA, caffeoyl-CoA and feruloyl-CoA) and aliphatic amines (putrescine, spermidine, agmatine) or aromatic amines (tyramine, tryptamine, and serotonin) (Bassard et al., 2010; Peng et al., 2016) and also represent the group of important phytoalexins involved in biotic and abiotic stress responses (Tanaka et al., 2003; Zeng et al., 2020b). In this study, the biosynthesis of flavonoids, including flavonoid C/O-glycosides, flavonol, flavone and anthocyanins, was significantly up-regulated in K69 compared to G72 (Supplementary Figure 8 and Supplementary Table 6). In addition, we also noted that the precursors of PAs, including L-phenylalanine, cinnamic acid, L-tyrosine, L-arginine and *p*-coumaric acid, were significantly decreased in the K69 line compared with the G72 line under PM infection (36–168 hpi) (Figures 3A–E). However, compared with the pattern of precursors of PAs, a significant elevation of PA levels was identified in the K69 line; among them, aliphatic PAs showed constitutive accumulation while aromatic PAs showed induced accumulation in qingke resistance to PM (Figure 3F).

When investigating differences in the metabolic responses to PM stress between qingke (K69) and barley (WDM00496), we observed that flavonoids were also notably increased in the two qingke varieties, while PAs, especially aromatic PAs, showed no significant changes in barley under PM treatment (Supplementary Table 10). The level of aromatic PAs was significantly induced at up to 30.0-fold in qingke when infected with PM (Supplementary Table 10), and this result was consistent with the above analysis (Figure 3F). Overall, these results indicated that a specific strategy existed in qingke to resist PM stress by inducing the accumulation of aromatic PAs.

To further validate these results, we used the PM-susceptible qingke (G72) that had been sprayed with two aromatic PAs for inoculation with PM. The results showed that the PM sporulation area in the aromatic PA-treated plants were much smaller than those in the blank control plants (Supplementary Figure 9). Therefore, the biosynthesis and regulation of aromatic PAs in qingke is interesting and will be further explored.

### *N*-Acyltransferase Genes Responsible for the Biosynthesis of Aromatic Phenolamides

**FIGURE 4:**
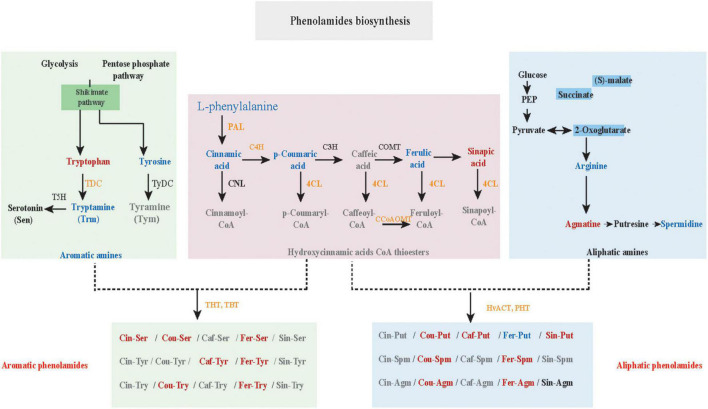
Differently expressed genes (DEGs) and accumulated metabolites (DAMs) from the pathway of phenolamides (PAs) biosynthesis in K69 and G72 lines of qingke during powdery mildew infection. The orange or green capital letters represented the significant up-regulated or down-regulated genes in K69 when compared to the G72 line. The expression level of these DEGs and the corresponding full gene name were listed in Supplementary Table 9. Red or blue lower case indicated markedly accumulated or reduced metabolites between the K69 and the G72 qingke line under powdery mildew stress.

Here, the genes and metabolites related to the PA biosynthesis were differentially regulated in the K69 and G72 qingke accessions (Figure 4). Specifically, upregulation of genes phenylalanine ammonia lyase (*PAL*), *p*-coumarate 3 hydroxylase (*C3H*), 4-coumarate: CoA ligase (*4CL*), *N*-hydroxycinnamoyl/benzoyltransferase (*HCT*), tryptamine hydroxycinnamoyl transferase (*THT*), tryptamine benzoyl transferase (*TBT*) and putrescine hydroxycinnamoyl transferase (*PHT*) related to the biosynthesis of amines, hydrocinnamic acids CoA and PA in the K69 line compared to the G72 line, was observed (Supplementary Table 9). The expression level of these nine genes from the PA biosynthesis pathway was verified and consistent with the transcriptomic data by qRT–PCR analysis (Supplementary Figure 10). Meanwhile, in the K69 line, aliphatic PAs showing the constitutive accumulation and the aromatic PAs with inducible accumulation were also observed in response to PM treatment (Figure 3F and Supplementary Table 6).

**FIGURE 5:**
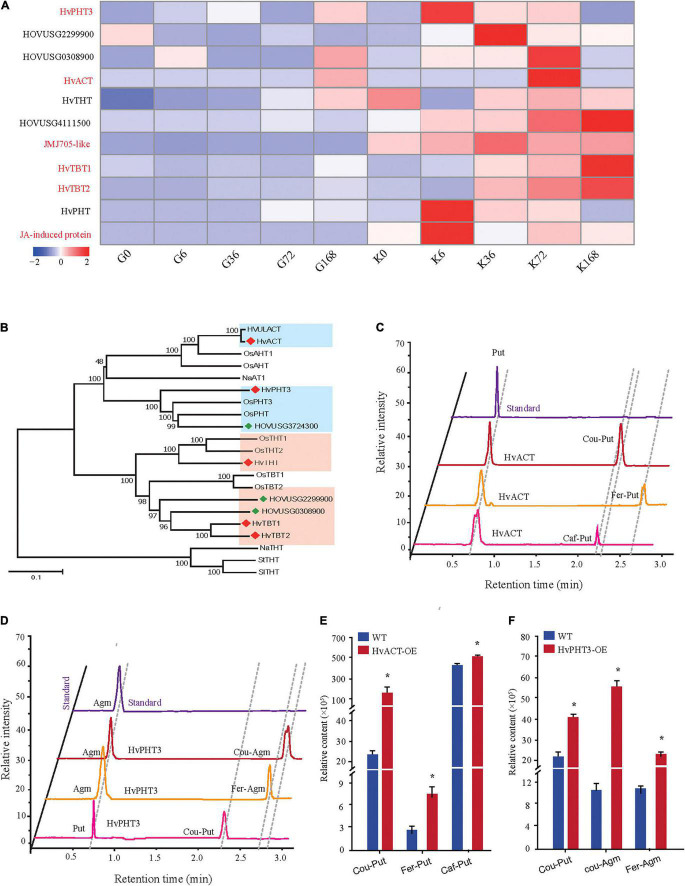
Functional characterization of *HvPHT3* and *HvACT*. **(A)** Expression pattern of PAs biosynthesis-related *N*-acyltransferases, JMJ705 and JA-induced protein in K69 and G72 lines during powdery mildew treatment. **(B)** The phylogenetic tree was built. BAHD proteins and five candidate genes sequences in phylogenetic tree were shown in Supplementary Table 2. **(C)** Validation of *HvACT* function by *in vitro* enzymatic activity. **(D)** Validation of *HvPHT3* function by *in vitro* enzymatic activity. **(E)** Bar plots for the content of Cou-Put (coumaroyl putrescine), Fer-Put (feruloyl putrescine) and Caf-Put (caffeoyl putrescine) in *HvACT* overexpressing tobacco and wild-type tobacco. **(F)** Bar plots for the content of Cou-Put (coumaroyl putrescine), Cou-Agm (Coumaroyl agmatine) and Fer-Agm (feruloyl agmatine) in *HvPHT3* overexpressing tobacco and wild-type tobacco. Data indicate mean value ± standard deviation (SD) with three replicates. * Indicates significant difference (*P* < 0.05, according to *t*-test). *HvPHT3*: *HOUSG2299900*; *HvACT*: *HOVUSG5340700*; *HvTHT: HOVUSG1220400*; *JMJ705*-*like*: *HOVUSG0811000*; *HvTBT1*: *HOVUSG0308500; HvTBT2*: *HOVUSG0309600*; *HvPHT*: *HOVUSG3724300*; *JA-induced protein*: *HOVUSG1730700*.

Phenolamides are secondary metabolic products acetylated by *BAHD N*-acyltransferases (Peng et al., 2016). To investigate the genes encoding *BAHD N*-acyltransferases responsible for the synthesis of PAs during PM infection, we analyzed the expression changes of all annotated *BAHD N*-acyltransferases in both qingke lines using FPKM values from RNA-seq. Interestingly, nine *N*-acyltransferase genes (*HvPHT3*, *HOUSG2299900*, *HOUSG0308900*, *HvACT*, *HvTHT*, *HOUSG4111500*, *HvTBT1*, *HvTBT2* and *HOUSG3724300*) had markedly higher expressions in K69; however, there was no significant change in the G72 line in response to PM stress (Figure 5A). Furthermore, the correlation analysis indicated that the expression levels of *HvTBT1*, *HvTBT2*, *HvPHT3*, *HvACT* and *HvTHT* were positively correlated with the PAs content (Pearson correlation coefficient (PCC) values > 0.6, *p* < 0.01) (Supplementary Table 11). To explore spatiotemporal expression of candidate genes, we collected samples at different stages from different parts of qingke and carried out PCR analysis. The results showed that *HvACT*, *HvTHT*, *HvTBT1* and *HvTBT2* were predominantly expressed in the roots while *HvPHT3* was expressed in the panicles and seeds (Supplementary Figure 11 and Supplementary Table 12). The phylogenetic analyses showed that proteins encoded by *HvPHT3*, *HvACT*, *HvTHT*, *HvTBT1* and *HvTBT2* were grouped into distinct *BAHD N*-Acyltransferases, which are known to catalyze the *N*-acylation of PAs, such as putrescine, tyramine and tryptamine, *in vitro* (Figure 5B) (Peng et al., 2016; Zeng et al., 2020b). Taken together, these results suggest that the five genes were identified as candidate genes participating in the biosynthesis of PAs.

To characterize the putative function of the five candidate genes, we performed an *in vitro* enzymatic assay using recombinant proteins in *E. coli* strain BL21 and *in vivo* transient expression in *N. benthamiana*. *HvACT* and *HvPHT3* exhibited strong acyl activities with aliphatic amines, such as putrescine and agmatine (Figures 5C–F and Table 1). In contrast, *HvTBT1* and *HvTBT2* mainly displayed strong activities on aromatic amines, such as tyramine, tryptamine, and serotonin, and weak activity on aliphatic amines (putrescine) (Supplementary Figure 12 and Table 1). These results suggest that the two genes *HvTBT1* and *HvTBT2* participate in the biosynthesis of aromatic PAs in qingke. Furthermore, to determine whether the two *N*-acyltransferase genes (*HvTBT1* and *HvTBT2*) responsible for aromatic PA biosynthesis were located in qingke-barley differentiation regions (The fixation index (*Fst*) between pairwise groups was calculated). First, we aligned the sequences of these two genes to the qingke reference genome (Zeng et al., 2015) used in a previous study of qingke-barley population differentiation. *HvTBT1* and *HvTBT2* are located at ∼55.68 and 473.55 Mb on chromosome 4, respectively. Then, through an alignment of chromosomal locations with the reported population differentiation regions, the two genes were exactly located in the qingke-barley population differentiation (Zeng et al., 2020b; Supplementary Figure 13). These results demonstrated that the aromatic PA synthesis pathway was naturally selected or artificially domesticated in qingke.

**TABLE 1:** Kinetic parameters of *HvACT*, *HvPHT3*, *HvTBT1* and *HvTBT*.

Substrate	*HvACT*			*HvPHT3*			*HvTBT1*			*HvTBT2*		
	*Km* (μM)	*Kcat* (S^–1^)	*Kcat/Km*	*Km* (μM)	*Kcat* (S^–1^)	*Kcat/Km*	*Km* (μM)	*Kcat* (S^–1^)	*Kcat/Km*	*Km* (μM)	*Kcat* (S^–1^)	*Kcat/Km*
**Donors^1^**												
*p*-Coumaroyl -CoA	104.9 ± 26.3	1.04 ± 0.13	0.010	105.1 ± 34.2	3.38 ± 1.20	0.032	ND	ND	ND	ND	ND	ND
Caffeoyl-CoA	483.5 ± 63.2	0.175 ± 0.01	0.001	ND	ND	ND	ND	ND	ND	ND	ND	ND
Feruloyl-CoA	161.3 ± 36.5	0.736 ± 0.02	0.005	320.3 ± 65.2	1.41 ± 0.13	0.004	224.9 ± 85.2	2.25 ± 0.58	0.01	381.7 ± 98.3	1.52 ± 0.62	0.001
**Acceptors^2^**												
Serotonin	ND	ND	ND	ND	ND	ND	9.68 ± 2.64	2.31 ± 0.85	0.239	46.0 ± 15.8	2.57 ± 1.02	0.056
Tryptamine	ND	ND	ND	ND	ND	ND	15.7 ± 8.25	0.69 ± 0.17	0.044	45.9 ± 10.6	2.94 ± 0.93	0.064
Tyramine	ND	ND	ND	ND	ND	ND	13.28 ± 5.7	1.11 ± 0.24	0.084	11.8 ± 5.9	2.56 ± 0.39	0.056
Putrescine	7.11 ± 2.14	2.51 ± 0.31	0.353	16.47 ± 2.15	3.18 ± 0.56	0.193	ND	ND	ND	11876 ± 425	1.52 ± 0.56	0.0008
Agmatine	ND	ND	ND	21.35 ± 5.37	2.10 ± 0.21	0.099	ND	ND	ND	ND	ND	ND

*^1^Amides (100 μM) as the acyl acceptor.*

*^2^Feruloyl-CoA donor (50 μM) as the acyl donor.*

*ND, not detected due to the low enzyme activity.*

### Methyl Jasmonate-Induced Demethylation and Promotion of the Biosynthesis of Phenolamides

**FIGURE 6:**
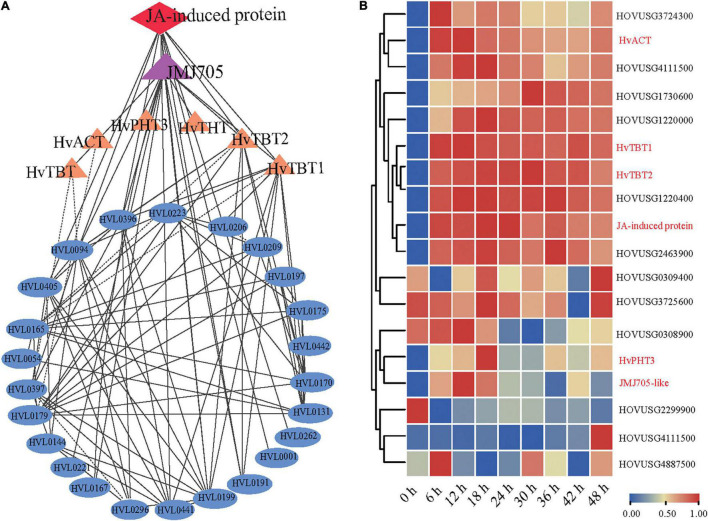
*JMJ705-like* and JA-induced proteins are associated with the biosynthesis of PAs. **(A)** Co-expression network among PAs, *N*-acyltransferases, JMJ705 and JA-induced protein using cytoscape software. Metabolites, genes and protein were shown in oval, triangle and diamond, respectively. The lines indicate the value of correlation coefficient for each correlated pairs greater than 0.6. **(B)** Hierarchical clustering analysis of PAs pathway-related genes expression levels based on qRT-PCR data. The gene expression level per row was normalized to 0 to 1. Expression levels were analyzed by quantitative RT-PCR. *HvACT*: *HOVUSG5340700*; *HvTBT1*: *HOVUSG0308500; HvTBT2*: *HOVUSG0309600*; *JA-induced protein*: *HOVUSG1730700*; *HvPHT3*: *HOUSG2299900*; *JMJ705*-*like*: *HOVUSG0811000*.

Coexpression network analysis is an important strategy to study the gene to gene or gene to metabolites interactions and their underlying functional correlations. To study the regulation of the PA biosynthesis pathway in qingke, we next used 13 PAs (Figure 4) as bait to screen highly correlated genes and proteins in our dataset based on the Pearson correlation coefficient (PCC). Our omics correlation data indicated that seven PA-related genes (*JMJ705-like* and six biosynthesis genes) and one protein (Jasmonate-induced protein) were strongly correlated with these PAs (PCC > 0.6, Supplementary Table 13). Furthermore, the correlation networks were constructed by calculating PCC values for the above identified gene/metabolite/protein pair (Figure 6A and Supplementary Table 13). Interestingly, coexpression network analysis revealed *JMJ705-like* protein, reported to be involved in MeJA-induced removal of H3K27me3 and activation of gene expression (Li et al., 2021), and jasmonate-induced protein were positively associated with PA biosynthesis in qingke (Figure 6A and Supplementary Table 13). Furthermore, the *JMJ705-like* and jasmonate-induced proteins displayed very similar expression patterns to the four *N*-acyltransferase genes during PM infection (Figure 5A). Taken together, these results imply that JA-mediated *JMJ705* may be involved in the biosynthesis of PAs by activating of the four *N*-acyltransferase genes responsible for PA synthesis.

**FIGURE 7:**
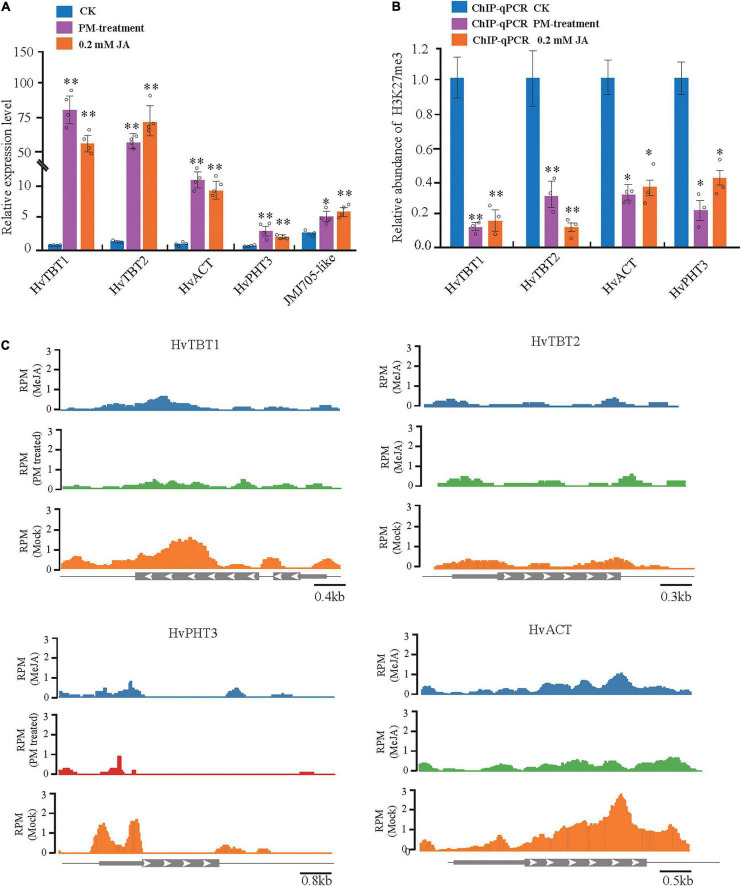
Analysis of MeJA-mediated (or PM-treated) regulation of the four *N*-acyltransferases. **(A)** The expression level of the four *N*-acyltransferases and JMJ705- like under control, powdery mildew (PM) and MeJA-treated plants. The expression values are represented as mean ± SD (*n* = 3). **(B)** H3K27me3 ChIP assays of the four JA- responsible *N*-acyltransferases treated with PM for 6 h and MeJA for 24 h. **(C)** H3K27me3 ChIP-on-chip data of the four JA-responsible *N*-acyltransferases in response to PM and MeJA treatment. Schematic representation of the gene structure. *P < 0.05, ^**^P < 0.001 by unpaired two-tailed Student’s *t*-test.

**FIGURE 8:**
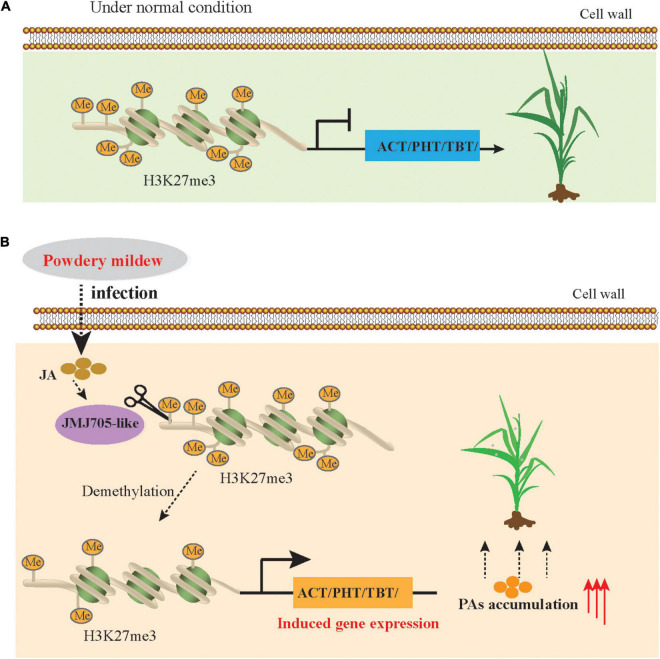
Simplified model of JMJ705 like-mediated regulation of expression of BAHD *N*-acyltransferases to improve powdery mildew resistance. **(A)** In the normal condition, H3K27me3 level is enriched in BAHD *N*-acyltransferases due to the absence of demethylases, and the these genes expressions are repressed. **(B)** Under powdery mildew stress condition, DNA demethylases (JMJ705) activity is increased duo to JA level rapidly synthesis, resulting in reduced H3K27me3 level, induced BAHD *N*-acyltransferases expression and increased the biosynthesis of PAs to against the powdery mildew pathogen.

To further investigate the regulation of PAs accumulation in response to PM infection, we treated 15-d-old seedlings of the K69 line with MeJA, one of the most potent elicitors of secondary metabolites and certain defense responses in plants (Zhan et al., 2020), for 0, 6, 12, 18, 24, 30, 36 and 48 h. qRT–PCR analyses indicated that the relative expression of *HvTBT1* and *HvTBT2*, mainly involved in aromatic PA biosynthesis, was increased ∼ 70.1- to 103.8-fold during the MeJA treatment (Figure 6B and Supplementary Table 14). Meanwhile, the expression levels of *HvACT* and *HvPHT3*, which were mainly involved in the biosynthesis of aliphatic PAs, were only ∼ 2.0 to 8.0 times higher in treated lines than in control lines (Figure 7A). These data suggested that MeJA treatment had a greater effect on the expression of the genes controlling aromatic PAs. In addition, the expression level of JMJ705 was also clearly upregulated by MeJA treatment (Figure 7A). Previous studies suggested that JMJ705 is closely associated with histone modifications, such as trimethylation of histone H3 lysine 27 (H3k27me3) (Li et al., 2013; Zhan et al., 2020). In this work, ChIP-seq and ChIP-qPCR assays from MeJA, PM and ethanol (mock) treatment of G72 line were performed. The results indicated that the H3K27me3 level of all four genes was significantly reduced under MeJA treatment (Figures 7B,C). Similar results were detected under PM infection (Figures 7B,C). These results reveal that the expression levels of genes related to PA biosynthesis were regulated by JMJ707-like-mediated removal of histone modifications and that this process was closely associated with MeJA signaling in response to PM stress. This result implies that the *JMJ705* gene may be involved in MeJA-mediated plant immune resistance, and the conservative mechanism may be widespread in Poaceae (Figure 8).

## Discussion

Plants have acquired diverse strategies to improve the resistance and tolerance of plants to abiotic and biotic stress, and one of the most important mechanisms is the hierarchical metabolic response that produces and accumulates diverse secondary metabolites (Fang et al., 2019; Wang et al., 2019). Recent advances in transcriptomics analyses have revealed that the expression level of genes changes in plants in response to PM stress (Amrine et al., 2015; Li Y. et al., 2019; Zhang et al., 2020), as one of the most devastating diseases in Triticeae crops, including wheat and barley. However, the underlying mechanism of plant resistance against PM remains unclear. Here, we used the multiomics data to comprehensively dissect metabolic pathways and characterize the functions and regulatory networks of these pathway-related genes in response to PM. Overall, our results revealed that PA biosynthesis is induced by PM stress in qingke. Specifically, the *BAHD N*-acyltransferase genes were upregulated directly by JA-mediated histone demethylation and eventually significantly improved PAs accumulation during PM infection in qingke (Figure 8).

Changes in some metabolites, such as flavonoids, flavonols and lignins, have been reported in previous studies (Fung et al., 2008; Molitor et al., 2011; Li et al., 2016). In barley, PM stress resistance was associated with carbohydrate metabolism flux redirection (Molitor et al., 2011). In wheat, phenylpropanoid biosynthesis and phenylalanine metabolism were the key pathways in response to PM infection (Fu et al., 2016). In addition, among the phenylpropanoid metabolites, flavonoid branches showed significant overaccumulation in qingke and barley. However, different types of metabolites were identified in this study. Under the PM treatment, the phenylpropanoid pathway was highly accumulated in the resistant qingke accession during PM infection. Omics data indicate that the biosynthesis of flavonoids, such as flavonoid C/O-glycosides, flavonol and flavone and anthocyanins, and PAs, especially aromatic PAs, induced accumulation in qingke in response to PM pathogens. More importantly, aromatic PAs, were identified only strongly inducibly in qingke, with no clear changes in barley.

Evidences have shown that JA plays an important role in the activation of defense gene expression and induction of resistance against pathogen infection (Back et al., 2001; Lee et al., 2001; Zhan et al., 2020). Previous studies showed that JMJ705, as a biotic stress-responsive H3K27me3 demethylase, was induced by JA to remove the H3K27me3 from defense-related genes for the activating of expression during pathogen infection (Li et al., 2013). Recently, JA also was demonstrated to be involved in controlling the accumulation of rice diterpenoids and enhancing the resistance of rice to bacterial blight by inducing the removal of H3K27me3 and gene activation (Zhan et al., 2020). Here, we have not only identified the levels of JA and JA-Ile to be rapidly induced as early as 6 hpi in the resistant qingke accession (Figure 2), but also, our data showed that MeJA/PM treatments upregulated *JMJ705-like* expression and reduced H3K27me3 levels from the four BAHD *N*-acyltransferases in the resistant qingke line (Figure 7). These results confirm that a timely JA signaling response plays crucial in the induction of *N*-acyltransferase genes expression in qingke.

## Conclusion

In conclusion, the integration of our transcriptomic, proteomic and metabolic data revealed that the metabolites within the phenylpropanoid pathway were mainly involved in the resistance response against PM. The induced overaccumulation of aromatic PAs was identified in a qingke-specific manner. Furthermore, a novel strategy for epigenetically controlling the biosynthesis of aromatic PAs was revealed in resistance to PM stress in qingke. Overall, this work not only revealed the regulatory mechanism underlying PM stress resistance associated with the accumulation of aromatic PAs but also provided a valuable PA-enriched qingke resource. These findings lay the foundation for promoting molecular design breeding and sustainable food in qingke.

## Data Availability Statement

The datasets presented in this study can be found in online repositories. The names of the repository/repositories and accession number(s) can be found below: National Center for Biotechnology Information (NCBI) BioProject database under accession number: PRJNA728483.

## Author Contributions

XZ and JL designed and supervised the project. QX, YW, CX, SH, and CZ participated in the material preparation, phenotypic identification, and DNA and RNA extraction. TT and SH conducted the metabolic profiling. CZ established the experimental system and organized the data. CX, CZ, and SH carried out the data analyses. CX wrote the manuscript. JL and XZ revised the manuscript. All authors discussed the results and commented on the manuscript.

## Conflict of Interest

TT was employed by the company Wuhan Metware Biotechnology Co., Ltd. The remaining authors declare that the research was conducted in the absence of any commercial or financial relationships that could be construed as a potential conflict of interest.

## Publisher’s Note

All claims expressed in this article are solely those of the authors and do not necessarily represent those of their affiliated organizations, or those of the publisher, the editors and the reviewers. Any product that may be evaluated in this article, or claim that may be made by its manufacturer, is not guaranteed or endorsed by the publisher.

## Footnotes

1
http://www.ccb.jhu.edu/software/hisat

2
http://www.yeastgenome.org/help/analyse/go-term-finder

3
https://cloud.metware.cn/#/user/login?redirect=%2F

4
http://www.anpel.com.cn/

5
www.igenebook.com

## Abbreviations

PAs: phenolamides
JA: jasmonic acid
MeJA: methyl jasmonate
hpi: hours post-inoculation
POD: peroxidase
SOD: superoxide dismutase
CAT: catalase
MDA: malondialdehyde
FPKM: fragments per kilobase of exon per million mapped reads
FC: fold change
PM: powdery mildew
ABA: abscisic acid
SA: salicylic acid
DEGs: differentially expressed genes
DAMs: differentially accumulated metabolites
DEPs: differentially expressed proteins
ChIP: chromatin immunoprecipitation.
