# Supplementary figures and images for: Resistance to Powdery Mildew in Qingke Involves the Accumulation of Aromatic Phenolamides Through Jasmonate-Mediated Activation of Defense-Related Genes
Source: Front Plant Sci. 2022 Jun 30;13:900345. doi: 10.3389/fpls.2022.900345 (PMC9280689; doi:10.3389/fpls.2022.900345)

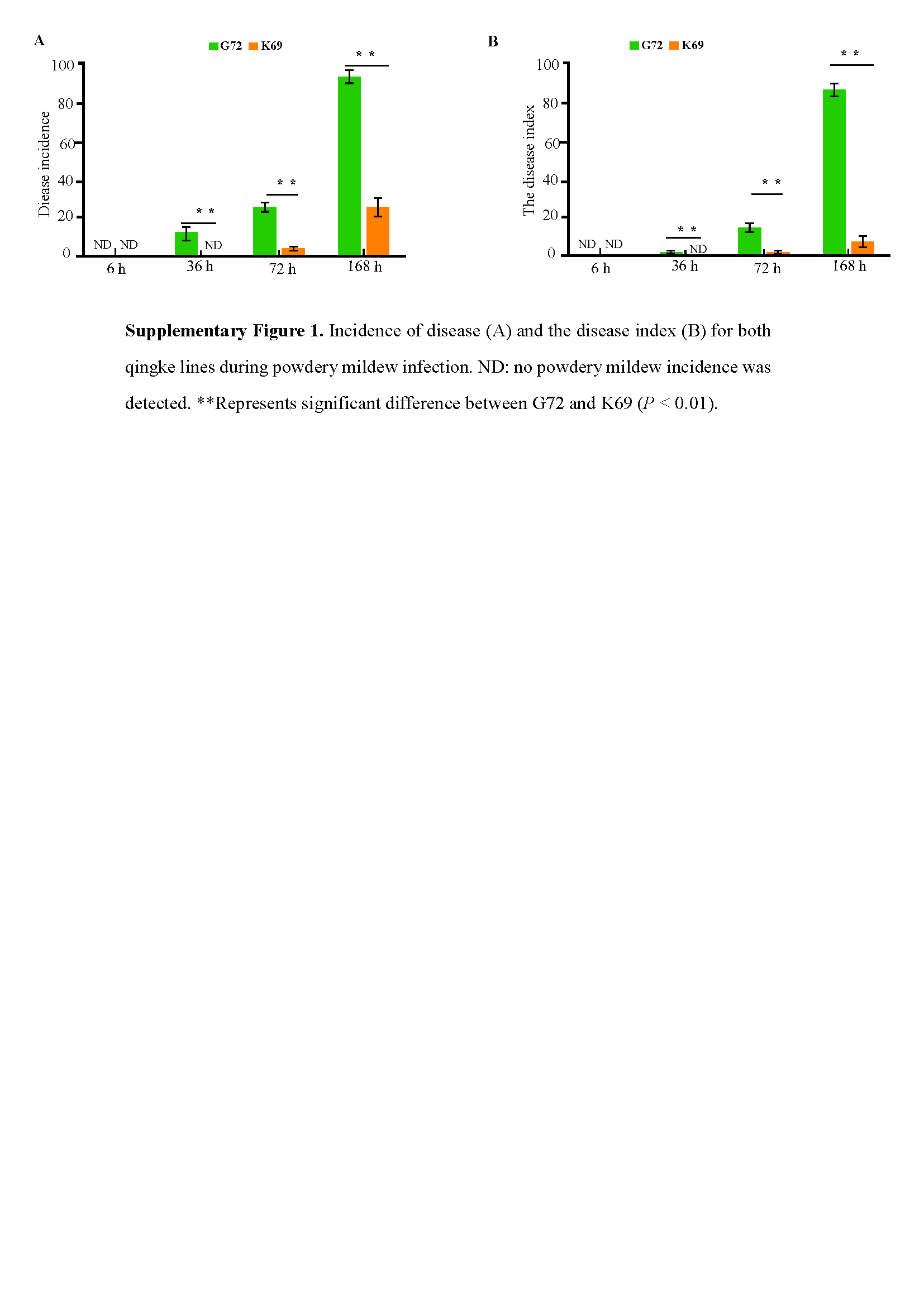

Supplement: Supplementary file 2 [file Image_1.TIF]

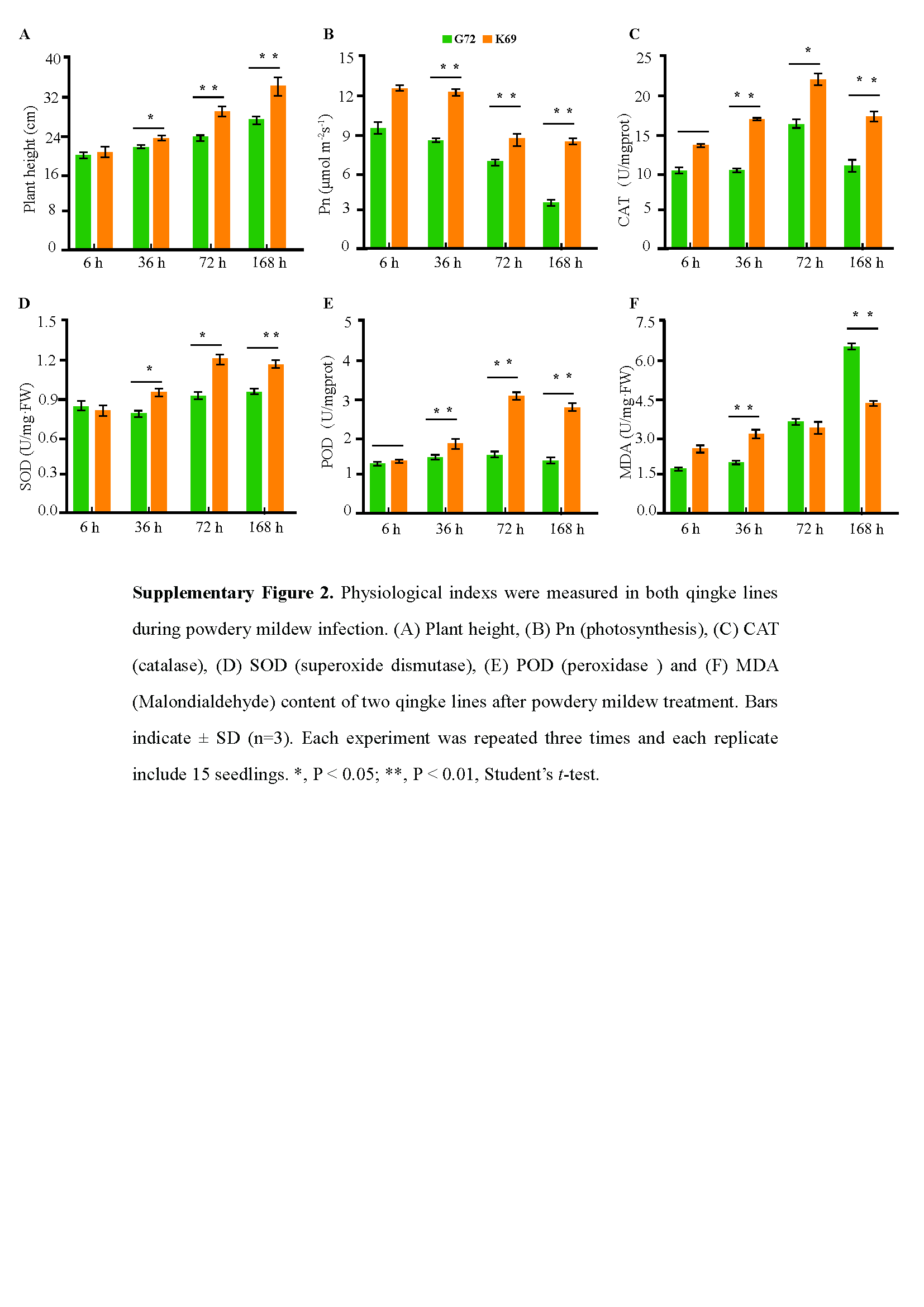

Supplement: Supplementary file 3 [file Image_2.TIF]

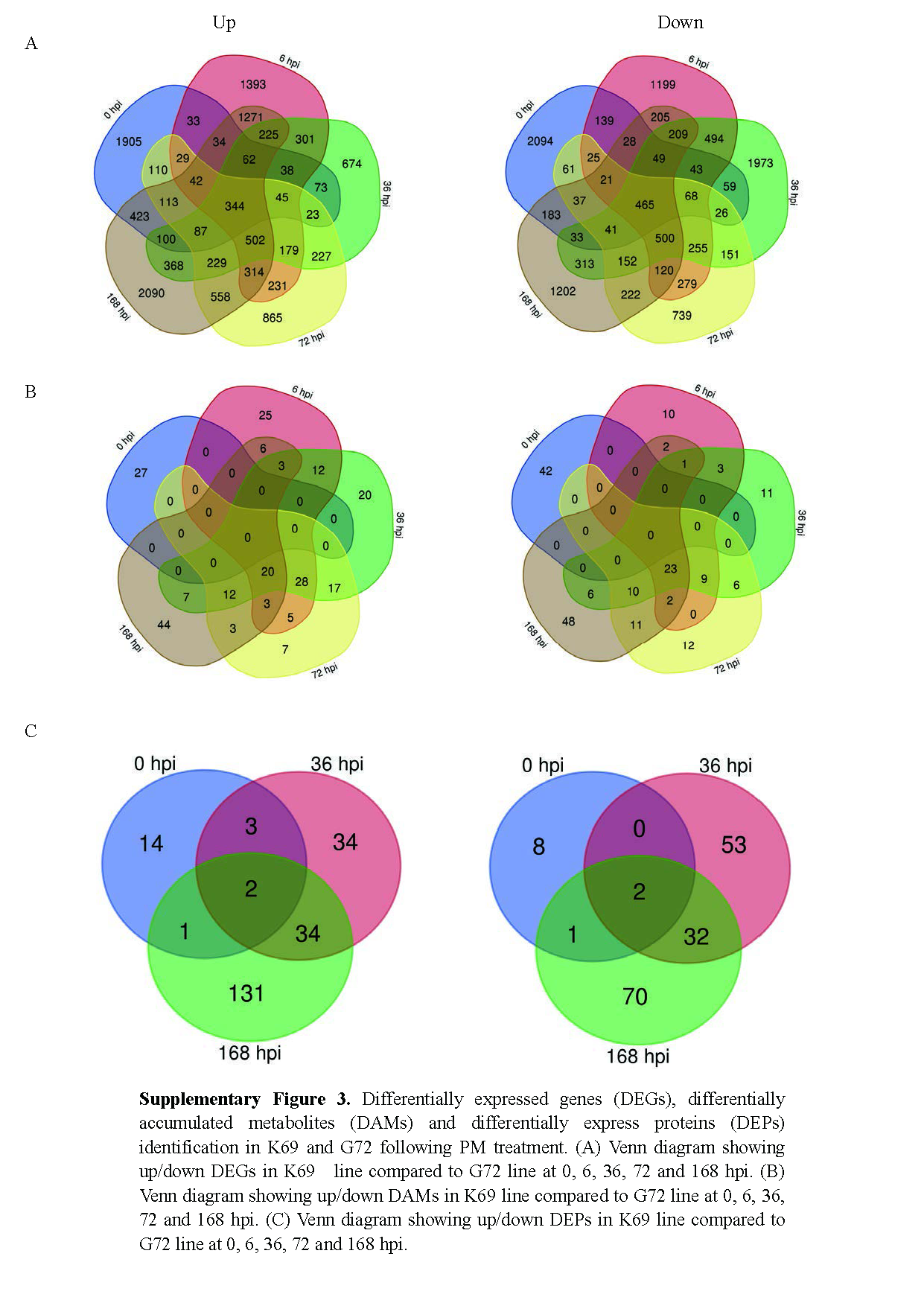

Supplement: Supplementary file 4 [file Image_3.TIF]

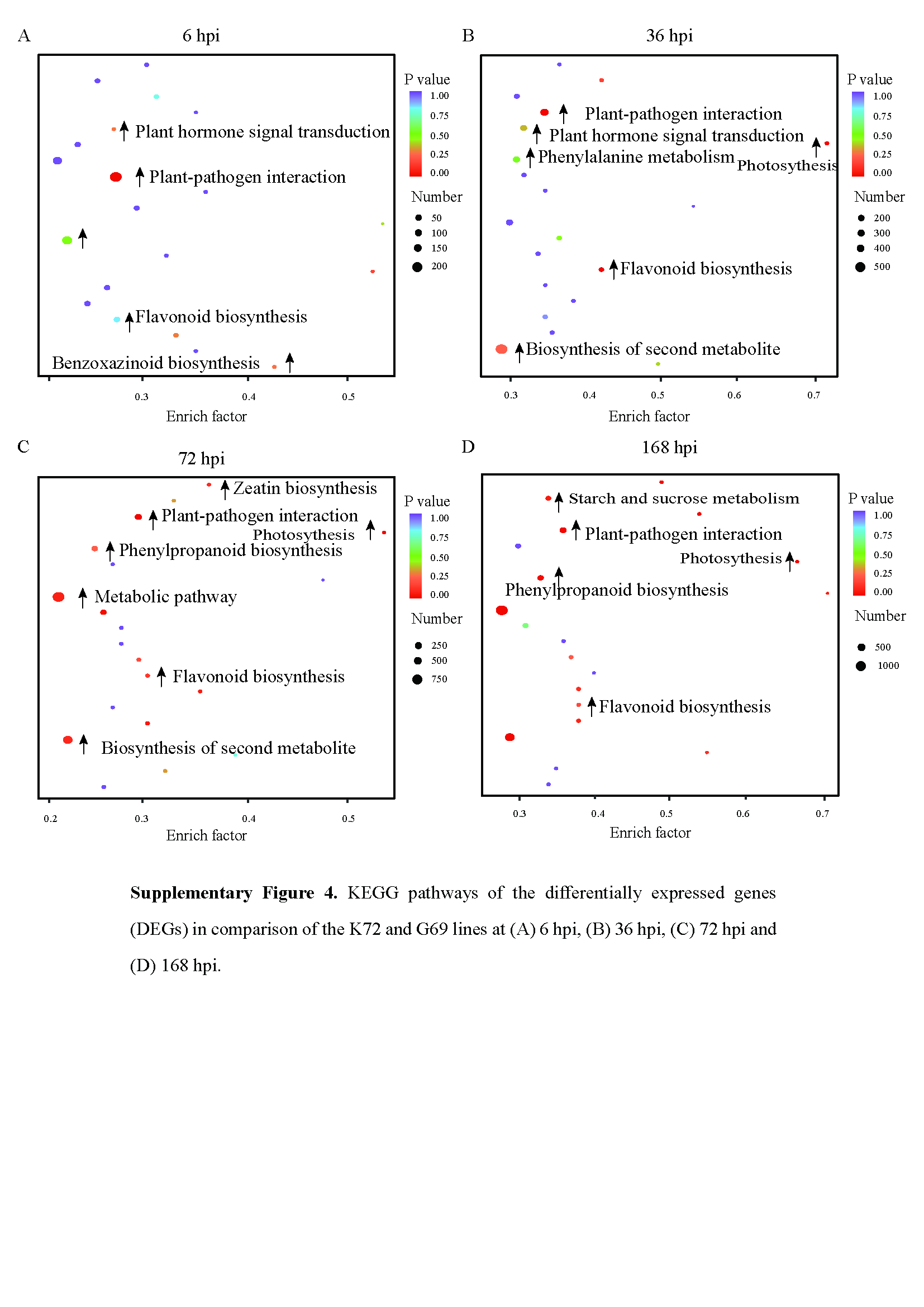

Supplement: Supplementary file 5 [file Image_4.TIF]

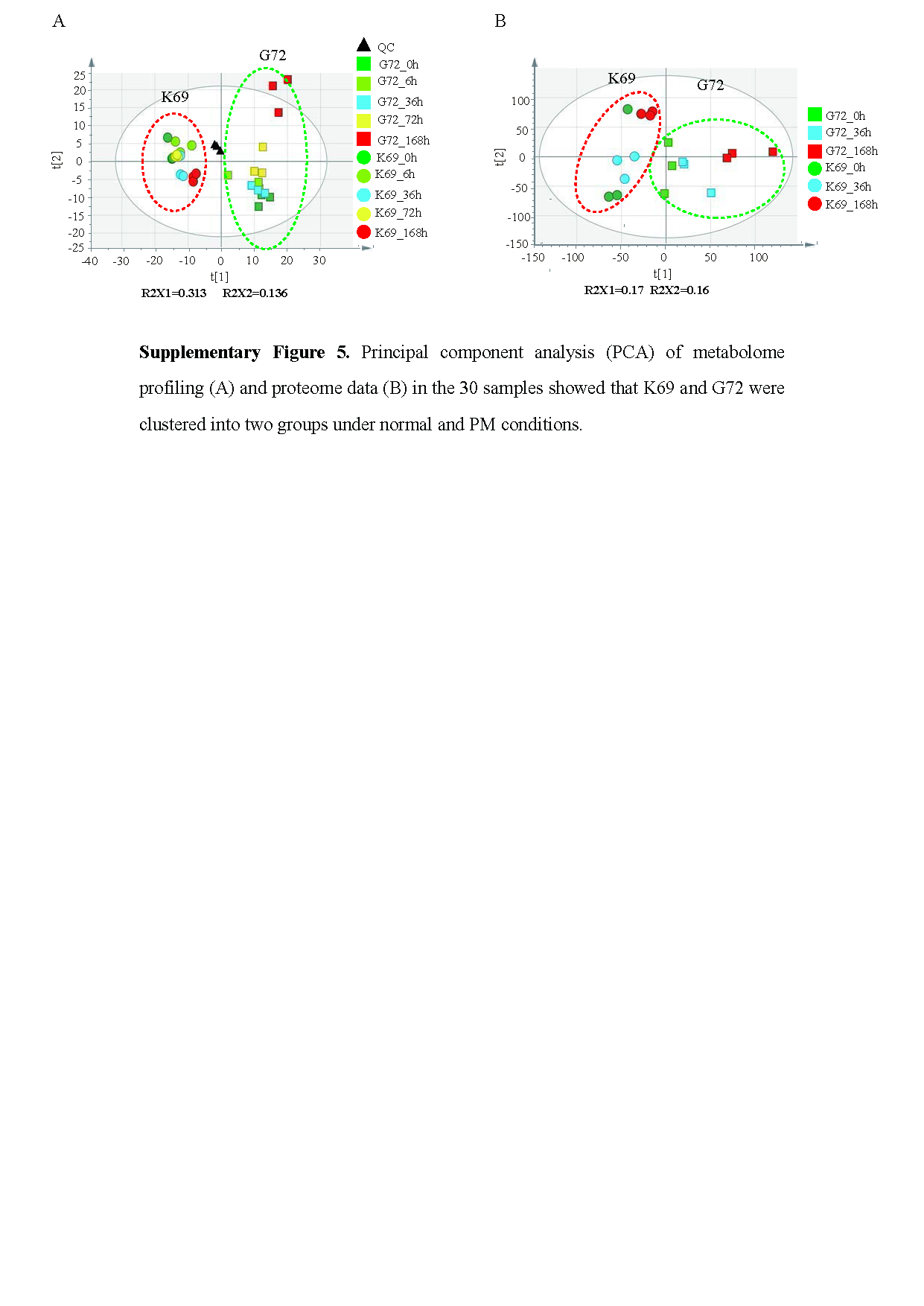

Supplement: Supplementary file 6 [file Image_5.TIF]

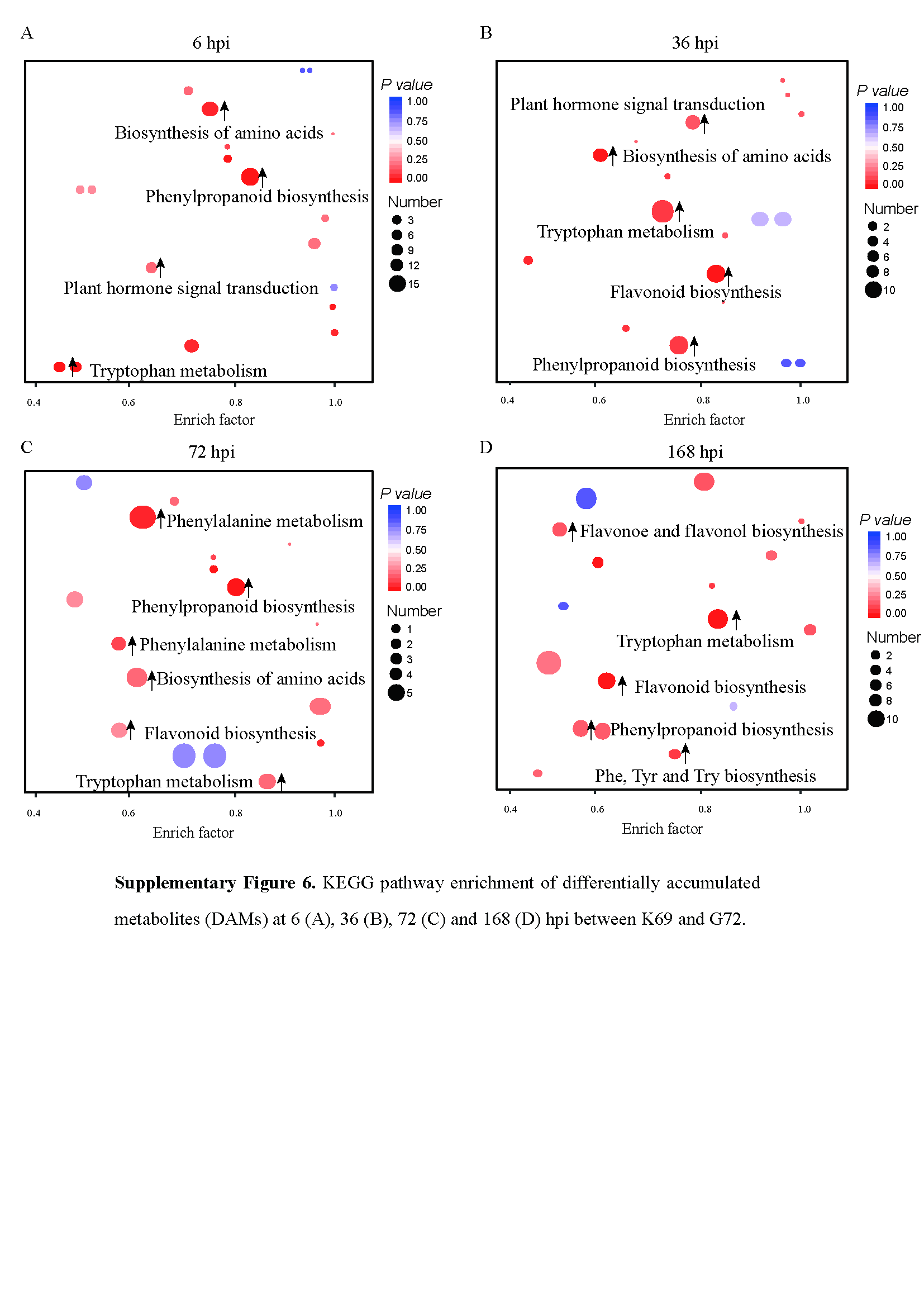

Supplement: Supplementary file 7 [file Image_6.TIF]

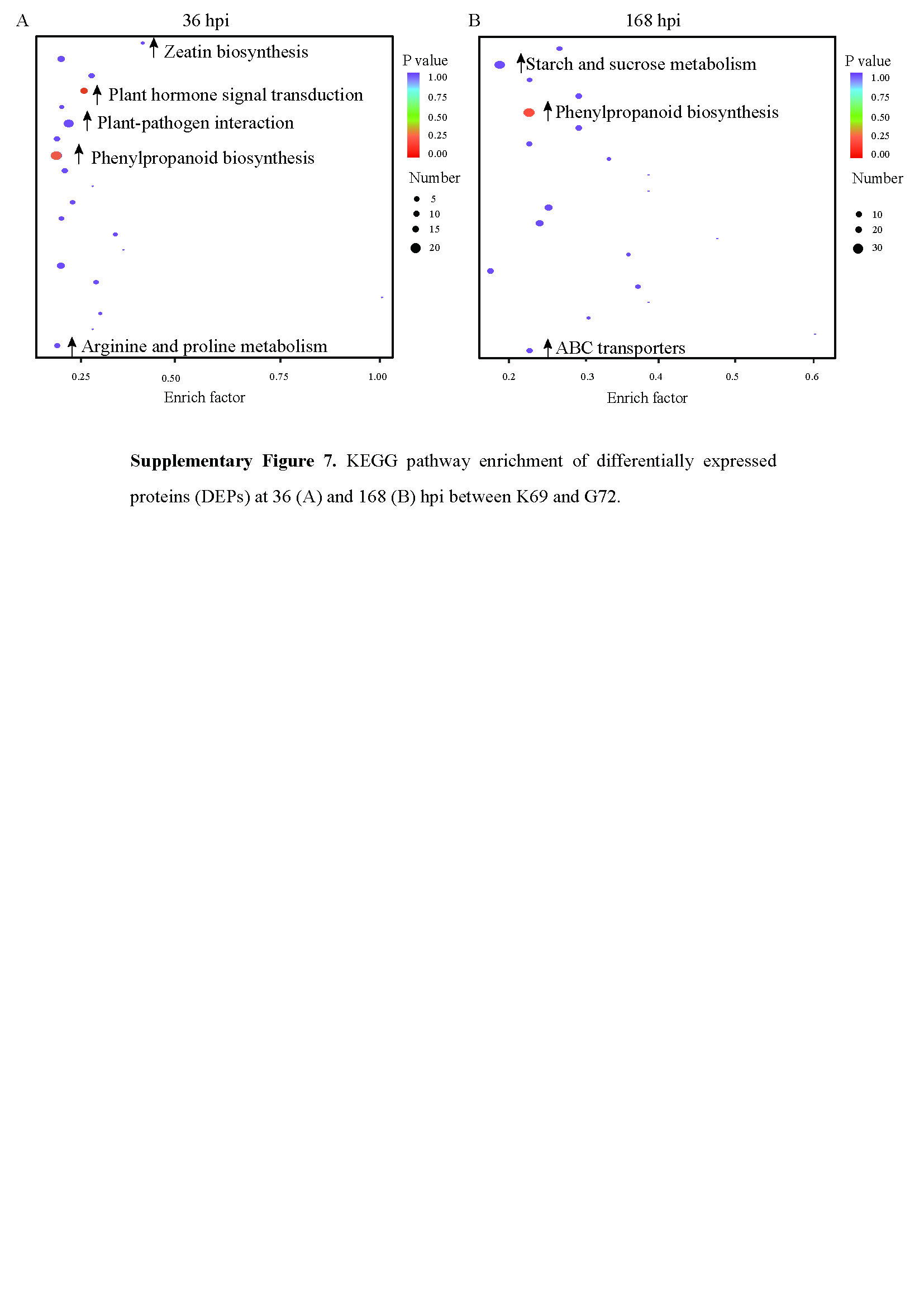

Supplement: Supplementary file 8 [file Image_7.TIF]

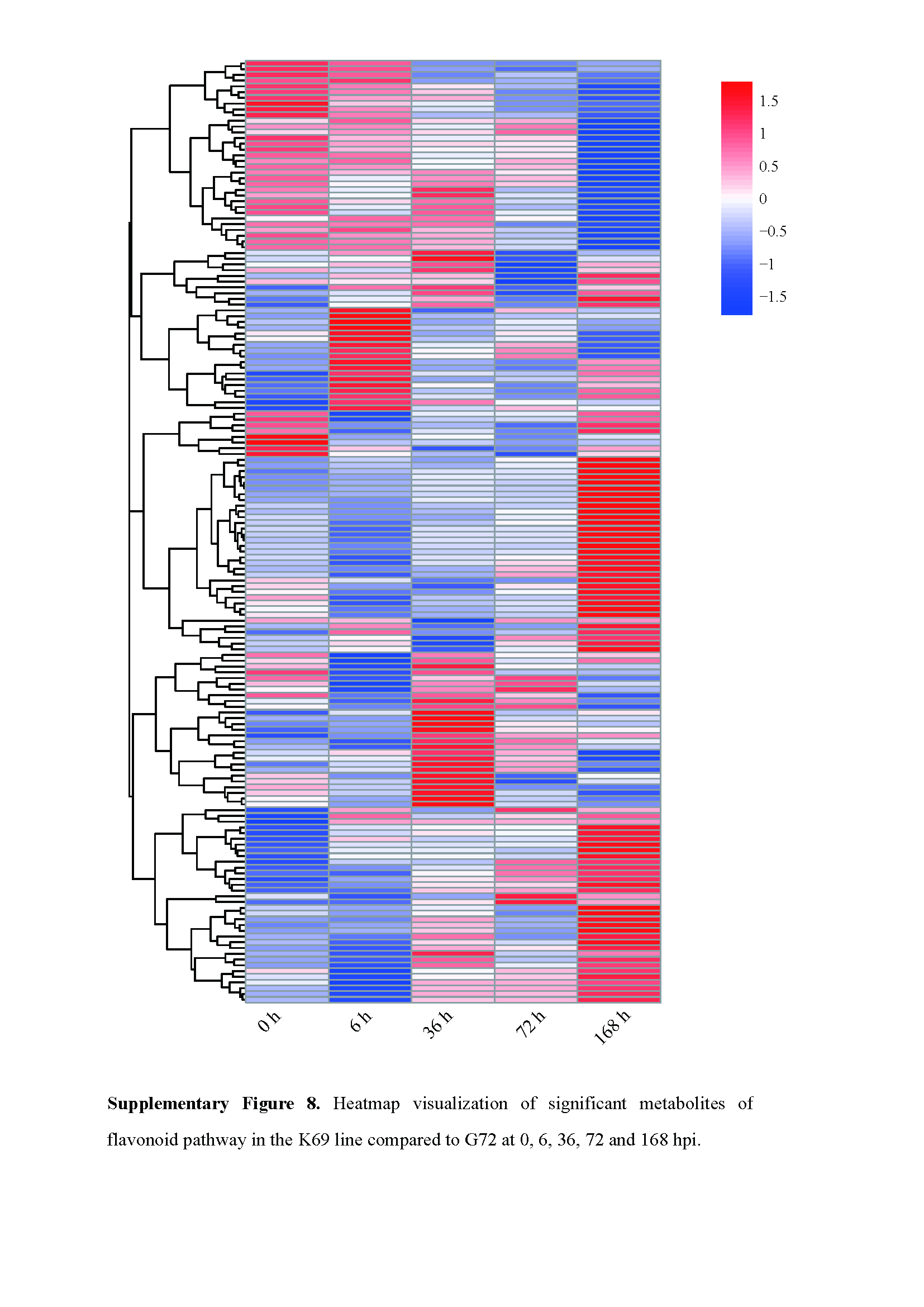

Supplement: Supplementary file 9 [file Image_8.TIF]

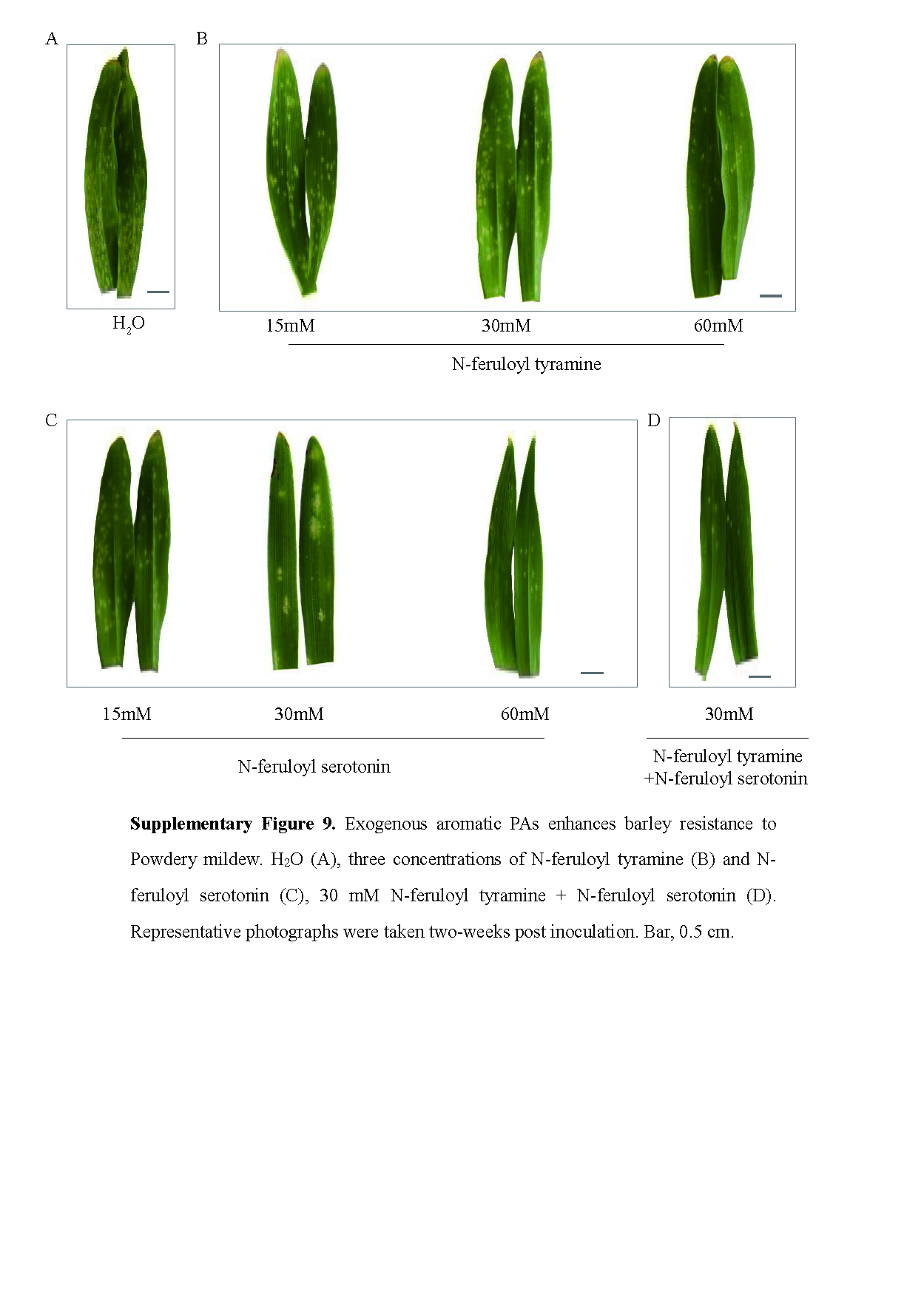

Supplement: Supplementary file 10 [file Image_9.TIF]

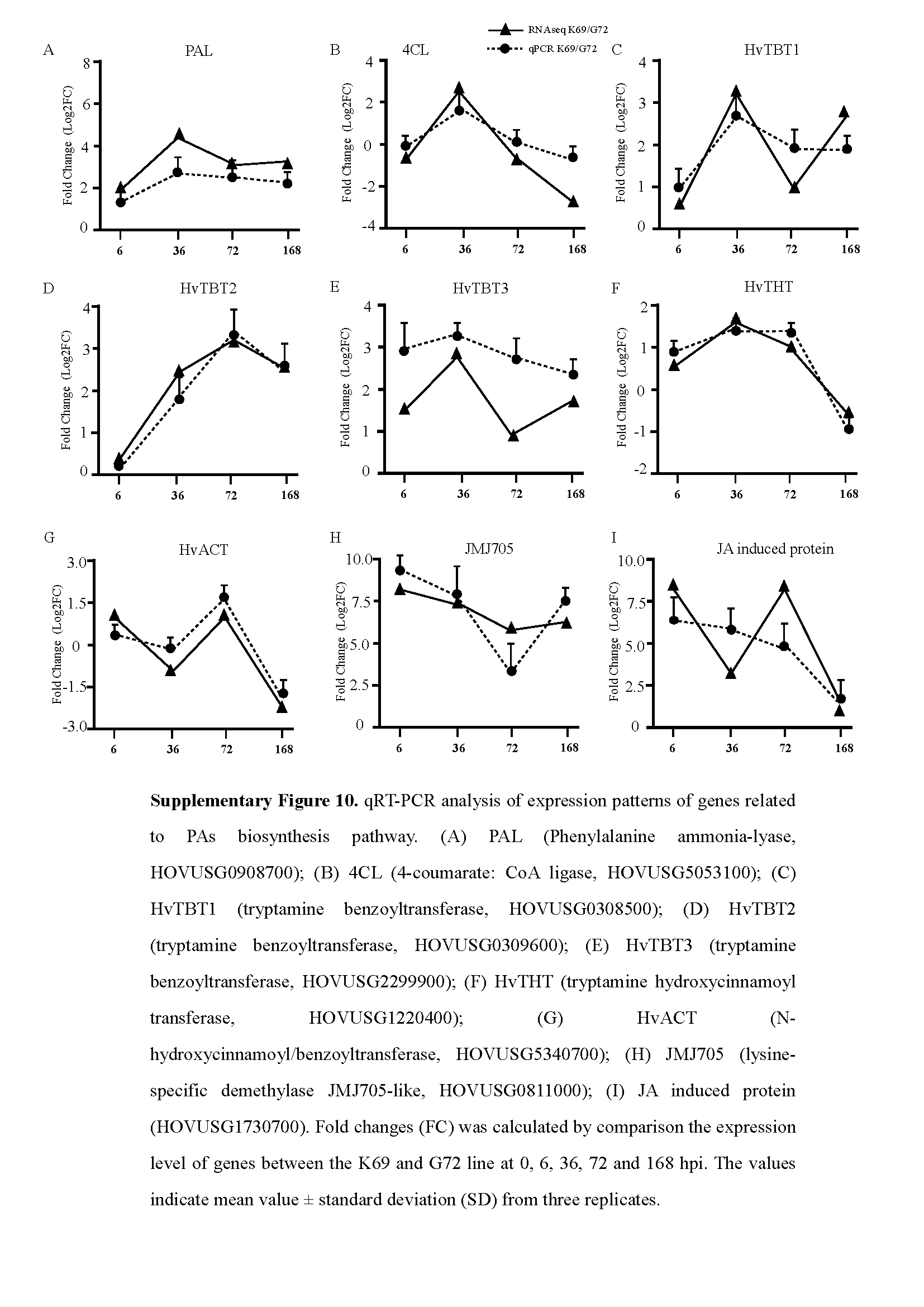

Supplement: Supplementary file 11 [file Image_10.TIF]

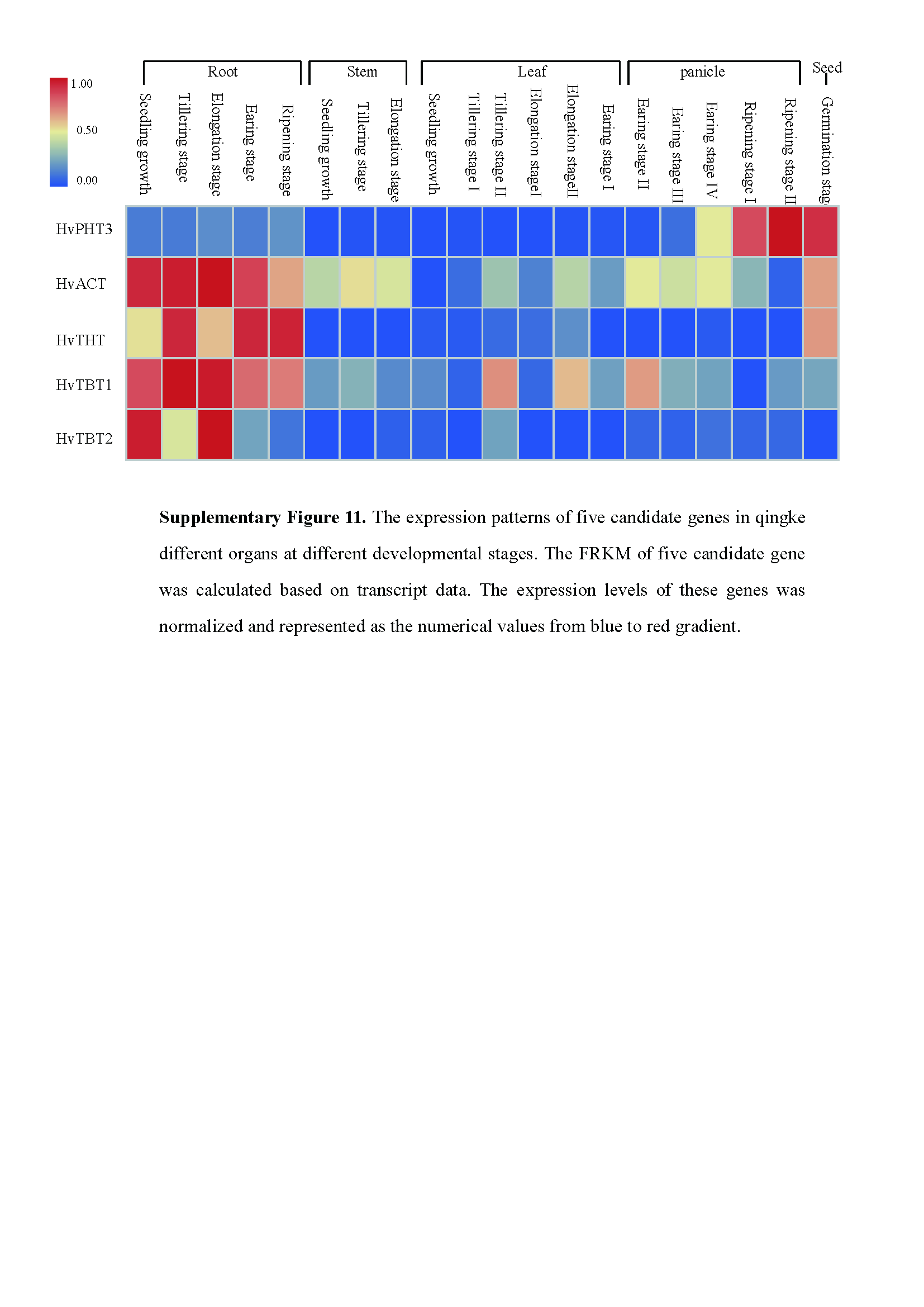

Supplement: Supplementary file 12 [file Image_11.TIF]

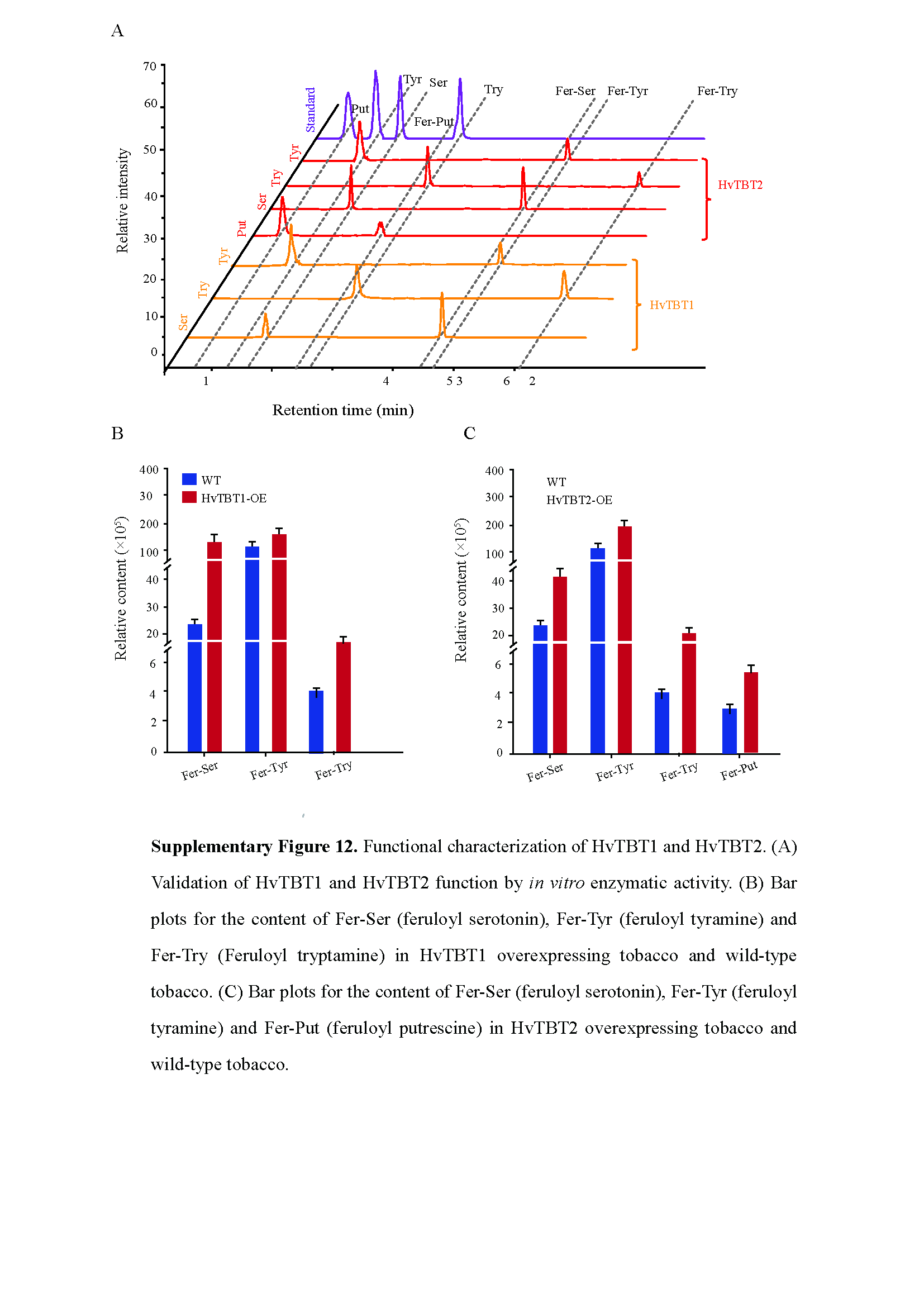

Supplement: Supplementary file 13 [file Image_12.TIF]

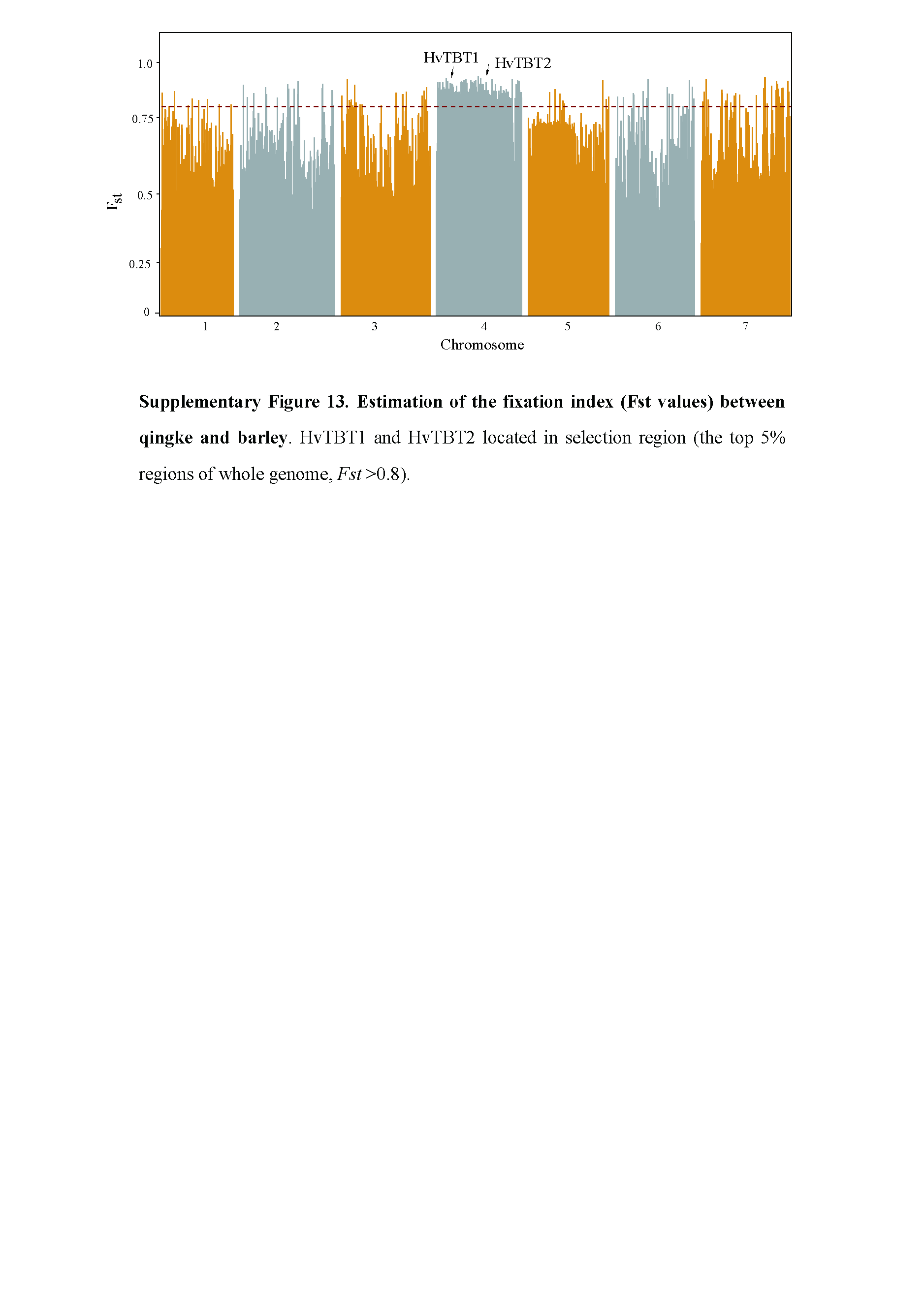

Supplement: Supplementary file 14 [file Image_13.TIF]
